# Nitrogen oxides (NO_*x*_) in nuclear fuel reprocessing off-gas streams: chemistry, control and impacts on radionuclide capture

**DOI:** 10.1039/d6ra08007c

**Published:** 2026-11-10

**Authors:** Jonathan Evarts, Brian J. Riley, Stuart Arm, Katherine R. Johnson, Craig Milroy, Joanna McFarlane, Krista Carlson, Randy Ngelale, Richard T. Mayes, Laura Arias Chavez, Alexander Wiechert, Harmony S. Werth, Joshua Turner, Chase C. Cobble, Jose Marcial

**Affiliations:** a Pacific Northwest National Laboratory Richland WA 99354 USA +1(509)372-7873 Jonathan.evarts@pnnl.gov; b Oak Ridge National Laboratory Oak Ridge TN 37830 USA; c National Nuclear Laboratory Warrington UK; d Materials Science and Engineering, University of Nevada Reno NV 89557 USA

## Abstract

Nitrogen oxides (NO_*x*_) in nuclear fuel reprocessing facilities present a challenging design landscape, due in part to their ability to degrade radionuclide capture materials, particularly at concentrations much higher than typical industrial cases. During used nuclear fuel dissolution in nitric acid, multicomponent off-gas streams evolve and include radionuclides such as ^129^I, ^131^I, ^3^H, ^14^C, ^85^Kr, and ^106^Ru, which require capture and proper disposition prior to discharging off-gases to the environment. Various methods and processes for radionuclide capture may be affected by NO_*x*_ generated during fuel dissolution and must be accounted for. Here, we review NO_*x*_ source generation, NO_*x*_ chemistry, methods for capture, sorbent material degradation pathways and several industrial nuclear fuel reprocessing plant industrial systems. It is observed that NO_*x*_ capture upstream of radionuclide beds is a common design theme in industrial reprocessing plants; however, options for NO_*x*_ capture sorbents are unproven in multicomponent, high concentration, off-gas streams.

## Abstract 2

Nitrogen oxides (NO_*x*_) in nuclear fuel reprocessing facilities present a challenging design landscape, due in part to their ability to degrade radionuclide capture materials, particularly at concentrations much higher than typical industrial cases.

## Graphical abstract

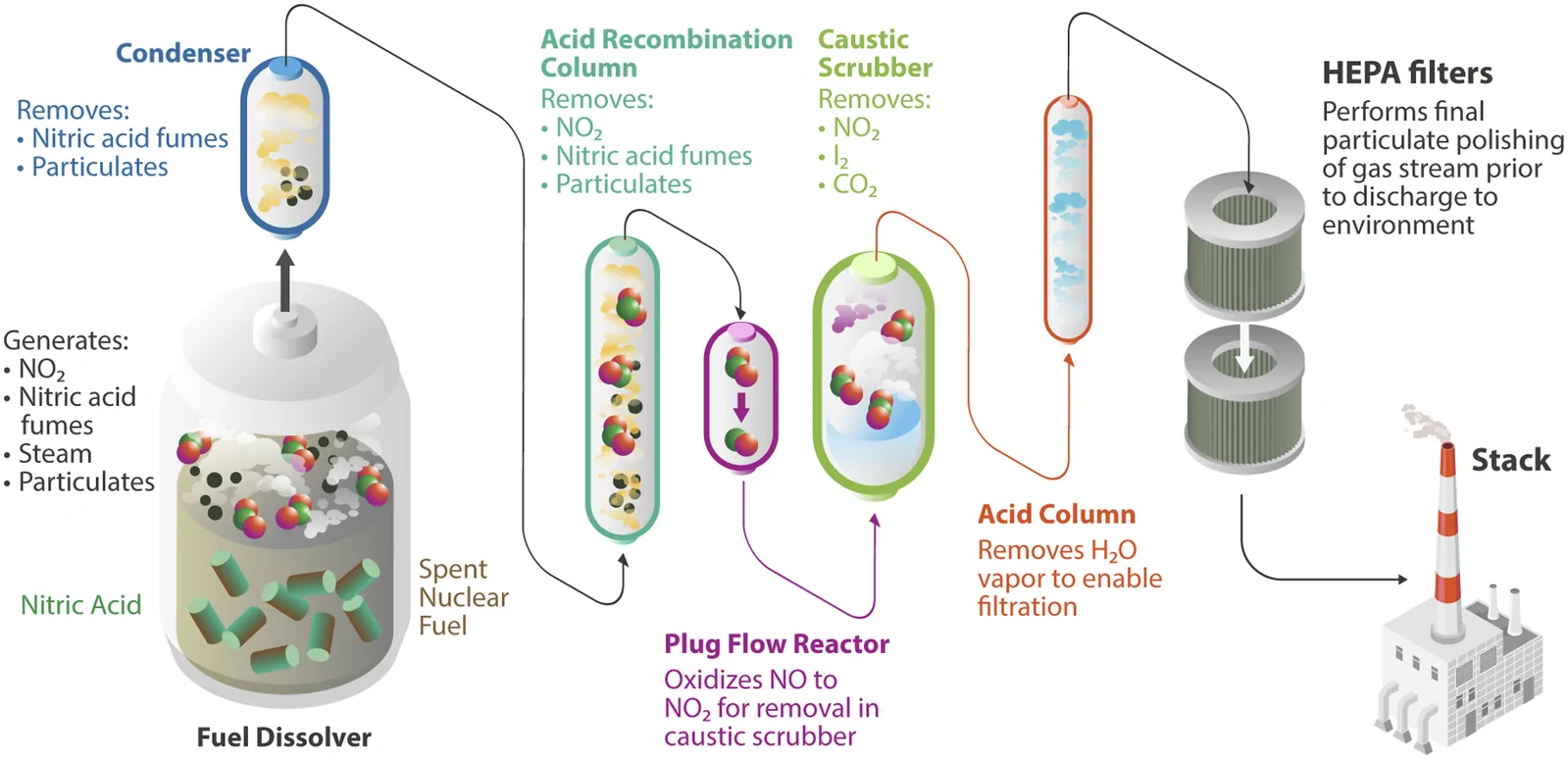

## 1 Introduction

Recent renewed interest in the United States Department of Energy (DOE) for recycling used commercial nuclear fuel (UNF) has generated a need for industrial-scale off-gas capture systems for volatile radionuclides, such as radioiodine (*i.e.*, ^129^I, ^131^I), tritium (^3^H), ^14^C, ^85^Kr, and ^106^Ru.^1^ Capture technologies are being explored with nonradioactive simulant streams in dynamic testing environments.^2–4^ These complex off-gas streams are further complicated by the presence of volatile and environmentally toxic chemicals added (*e.g.*, HNO_3_ in the PUREX process) or generated (*e.g.*, NO_*x*_) during fuel reprocessing efforts. In aqueous reprocessing, nitrogen oxides, primarily NO and NO_2_, are produced during fuel dissolution where mechanisms such as exposure-induced degradation reduce the performance of upstream radionuclide (*e.g.*, radioiodine) abatement technologies. These mechanisms are further enhanced by the addition of NO_2_ at higher concentrations, as used during advanced voloxidation.^5^

Over the past several decades, significant advancements in NO_*x*_ abatement technologies have been realized in well-developed, non-nuclear industrial applications such as heavy-duty diesel emissions and combustion flue gas^6–9^ but these methods and results are not directly transferable to nuclear-based aqueous reprocessing.^10–12^ For example, NO_*x*_ concentrations in industrial off-gases are typically on the order of ppm^13,14^ while typical NO_*x*_ concentrations in aqueous-based UNF reprocessing are on the order of several vol%.^15^ Similarly concentrations in the dissolver off-gas (DOG) can reach up to 20 vol%;^4^ however, this is highly dependent upon dissolver operations. In advanced voloxidation, NO_2_ feed concentrations can reach 50–75 vol% with NO as the majority off-gas species.^5,16^ The significant difference in NO_*x*_ concentration can have meaningful impacts downstream for radionuclide capture performance.

The presence of NO_*x*_ complicates radioiodine capture across both solid- and liquid-phase treatment systems. In solid sorbent beds, NO_*x*_ can alter sorbent speciation by deactivating reactive binding sites, and chemically degrade the capture material.^17–21^ Although alkaline solutions can efficiently scrub inorganic iodine, residual NO_*x*_ also reacts with the caustic solution to form nitrite and nitrate, increasing hydroxide consumption, chemical demand, and the volume and complexity of secondary liquid waste.^22–24^ ORNL studies show that exposure of reduced silver mordenite to NO-containing streams at 150 °C can sharply reduce iodine capacity, with reported losses of roughly 78–80% after short-term to multiweek exposures, while silver-functionalized aerogels show smaller, but still significant, NO_*x*_-dependent losses.^18,21^ Baskaran and co-workers similarly showed that NO_2_ aging changes bismuth-based iodine-capture chemistry.^25,26^ Thus, proper off-gas system design is crucial for effective capture of radionuclides during aqueous fuel reprocessing in the presence of these chemicals.

In addition to higher NO_*x*_ concentrations, other complications such as the presence of multiple radioactive species within the off-gas stream and secondary radioactive waste constraints create complications for robust and efficient engineering solutions.^2,4,24^ In general, a NO_*x*_ mitigation strategy that works well for relatively simple ppm-level NO_*x*_ streams, might not work for the voloxidation stream that has a much higher NO_*x*_ concentration and complex chemistries. An example of this is that a solid sorbent bed targeted at capturing NO_*x*_ could get quickly saturated while a titration-driven approach within liquid scrubber system could maintain a target pH within the solution.

While there are several international examples utilizing NO_*x*_ abatement technologies, it is essential that effective off-gas management be implemented to preserve radionuclide (*e.g.*, radioiodine) capture performance and maintain the required decontamination factors (DFs) imposed by the environmental regulations documented in 40 CFR 190. The definition of DF is provided by eqn (1), where *E* is the capture efficiency (mass fraction capture), *P* is bed penetration (mass fraction breakthrough) and *P* = 1 − *E*.1
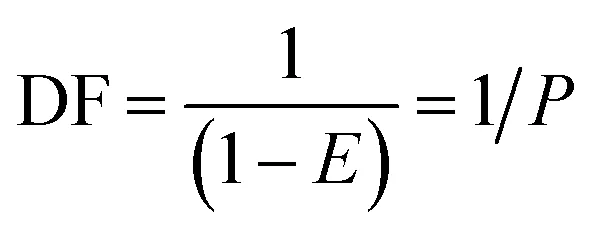


In general, NO_*x*_-abatement strategies may place NO_*x*_ capture upstream, in parallel with or downstream of iodine scrubbing (Fig. 1). Scrubbing NO_*x*_ upstream of the iodine solid sorbent bed will lead to notable increases in the sorbent bed operational lifetime and DFs, depicted by the green-line in Fig. 1(a); however, industrial plants typically employ hybrid arrangements, as shown in Fig. 1(b). Further, there is a potential that with no NO_*x*_ abatement, downstream noble gas capture could be affected, but there is little available information on the impact of NO_*x*_ on this parameter.

**Fig. 1:**
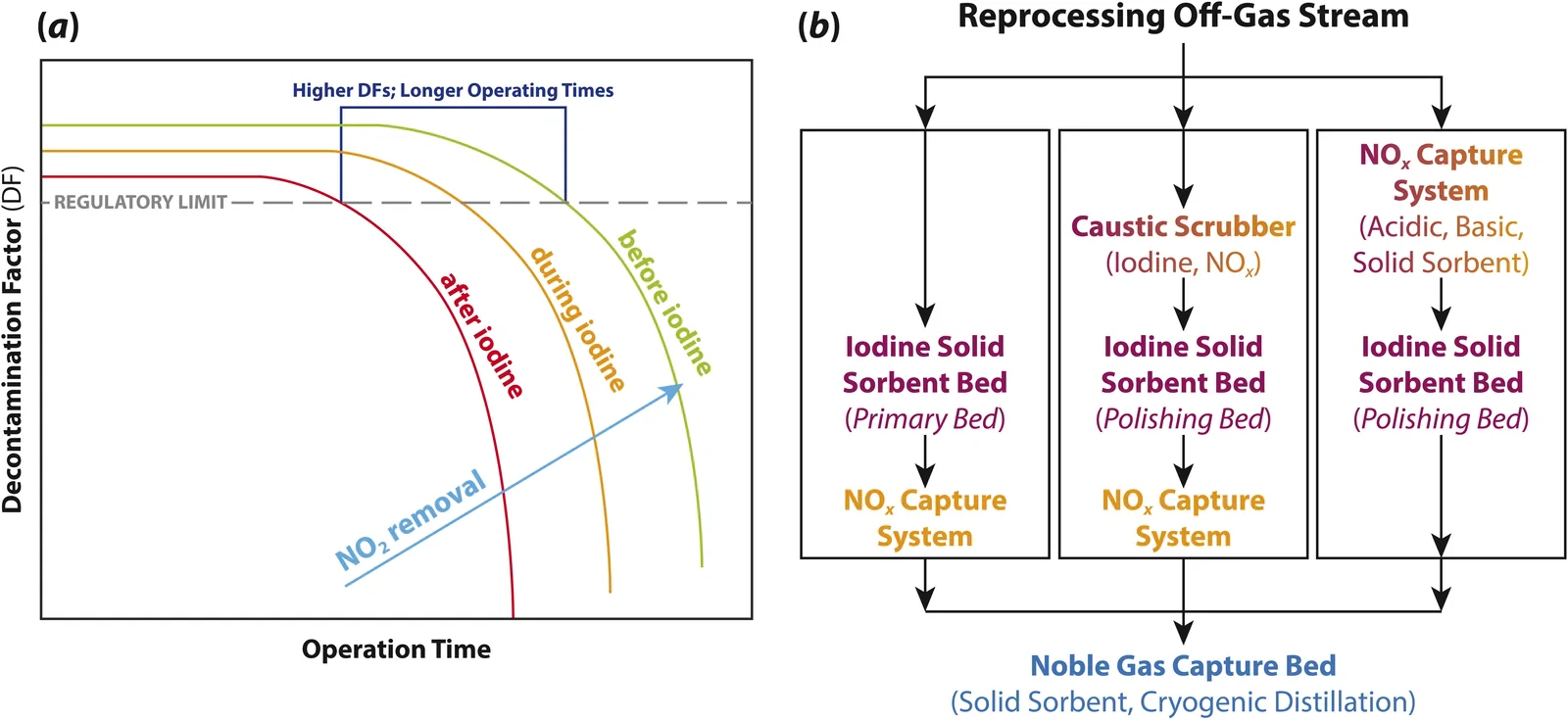
(a) Conceptual illustration of NO_2_ decontamination factors for NO_2_ removal before (upstream), during and after (downstream) iodine scrubbing. (b) Summary figure showing the options for NO_*x*_ capture in the off-gas treatment system.

The Thermal Oxide Reprocessing Plant (THORP) at the Sellafield site in Cumbria, England and the nuclear fuel reprocessing plant at La Hague, France use acid absorption or nitric-acid recombination to remove a substantial fraction of NO_2_ (residual NO is oxidized to the more readily scrubbed NO_2_ form) and a downstream caustic scrubber to capture the remaining NO_*x*_ together with inorganic iodine species. Thus, simultaneous caustic scrubbing can provide effective final polishing, but it consumes alkaline reagent and generates a comparatively large nitrate-, nitrite-, and radioiodine-containing liquid-waste stream. Rokkasho incorporates additional solid sorbent beds for final radionuclide capture.^27,28^ Conversely, placing NO_*x*_ removal only downstream of a solid iodine sorbent does not protect that material from NO_*x*_-induced aging. Maximizing NO_*x*_ removal before the principal solid iodine-capture bed should therefore reduce sorbent exposure, extend operational lifetime, and help preserve iodine decontamination performance, while a downstream scrubber or polishing bed may still be required for residual contaminants. Important uncertainties remain regarding species-specific decontamination factors for NO, NO_2_, and inorganic and organic iodine across individual and integrated treatment operations.

## 2 Regulatory drivers

In the U.S., several regulatory drivers guide the capture requirements for NO_*x*_. The release of radionuclides is subject to NRC (10 CFR 20) and EPA (40 CFR 190) regulations. DOE orders, *e.g.*, 458.1, to protect the public from radiation associated with DOE activities may also apply if the recycling facility is operated by DOE. Nuclear facilities are considered stationary sources and are subject to multiple regulations administered by the EPA, including the Clean Air Act (CAA), State Implementation Plan (SIP), and New Source Performance Standards (NSPS) on specific unit operations, Table 1. The CAA focuses on the total ambient concentration of NO_*x*_ over a given local area following the National Ambient Air Quality Standard (NAAQS) for NO_*x*_: 100 ppb h^−1^ and an annual average of 53 ppb.^29^ SIPs enforce state limits on NO_*x*_ and may vary across industries. In addition, SIPs are decided on a case-by-case basis. Typically, SIPs also include requirements for reporting and monitoring NO_*x*_ fumes that exit the facility. NSPS limits are dependent on the equipment type and size that are within a facility and are determined by performance data from the EPA. Further, other NSPS rules may apply if a nuclear facility uses captured NO_*x*_ fumes to regenerate nitric acid for additional fuel dissolution. In 2012, NSPS set a limit of 0.50 lbs NO_*x*_·per ton of 100% HNO_3_ produced, equivalent to approximately 0.23 kg NO_*x*_ per ton of HNO_3_, evaluated at a 30 days average emission rate.^30^

**Table 1:** Summary of US regulations for NO_*x*_ emissions^a^

Regulation	NO_*x*_ limit	Defined by	Applies to	Relevant regulation
Clean Air Act (CAA)	100 ppb h^−1^ or 53 ppb	NAAQS	Facility	40 CFR 50
New Source Performance Standards (NSPS)	Varies; 0.23 kg NO_*x*_ per ton HNO_3_	Performance data on unit type and size	Equipment	40 CFR 60 subpart Db, Dc, and G
State Implementation Plan (SIP)	Varies	BACT	Facility	40 CFR 51
New Source Review (NSR)	Varies	BACT/LAER	Facility	40 CFR 51.165–51.166

a BACT = Best Available Control Technology (used in the U.S. Clean Air Act); CFR = U.S. Code of Federal Regulations; LAER = Lowest Achievable Emission Rate (used in the U.S. Clean Air Act); NAAQS = National Ambient Air Quality Standards.

Stationary sites like nuclear facilities must have a Title V operating permit that outlines emission limits and monitoring requirements for emissions > 100 tons NO_*x*_·per year. In many cases, new or modified facilities would have to undergo a New Source Review (NSR) and may require incorporating a Best Available Control Technology, or BACT, with either catalytic or noncatalytic reduction techniques to enforce the lowest achievable emission rate (LAER) resulting in a desired emission offset. Given that there are no nuclear fuel recycling plants operating in the United States, any future fuel recycling operation and any other new nuclear facility will be subject to absorbing the cost to meet these criteria.

## 3 NO_*x*_ generation and speciation in aqueous-based UNF reprocessing

Although significant accomplishments in NO_*x*_ scrubbing have been achieved in the past several decades, the focus was distinctly based upon industrial off-gases where NO_*x*_ concentrations are typically in the ppm range and contain fewer contaminants.^31–33^ Aqueous reprocessing based on the Plutonium and Uranium Reduction Extraction (PUREX) process is the most common industrial scale process used to recover/recycle uranium and plutonium from UNF, and has been used for decades to reprocess UNF from light water reactors (LWRs), pressurized heavy-water reactors (PHWRs), boiling water reactors (BWRs), advanced gas-cooled reactors (AGRs), Magnox reactors, and fast reactors (FRs), among others.^34^

### 3.1 NO_*x*_ source chemistry

The “head end” stage of the PUREX process involves dissolving sheared (chopped up) UNF in concentrated nitric acid (4–9 M) at elevated temperatures (60–90 °C). The reaction between uranium dioxide (UO_2_) and nitric acid (HNO_3_) oxidizes uranium from +IV to +VI and produces soluble uranyl nitrate salts [UO_2_(NO_3_)_2_], as well as HNO_3_ reduction by-products. During UNF reprocessing, off-gas streams may comprise several vol% of NO_*x*_ and include other radioactive contaminants such as radioiodine, ^3^H, and ^14^C, which also must be captured for disposal.^5^

The mechanism of NO and NO_2_ production resulting from aqueous UNF reprocessing in HNO_3_ remains a field of ongoing study.^35,36^ Marc and coworkers^36^ have compiled a review of known literature in the area, and some general trends may be observed: (i) there are 7 distinct and potentially competing mechanisms; and (ii) of the 7 mechanisms, reactions yielding NO_2_ predominate with high HNO_3_ concentrations and NO with low concentration. Yet, there are two mechanisms that literature consensus indicates to be the key pathways, shown in eqn (2) and (3):

2 UO_2_ + 2.7HNO_3_ → UO_2_(NO_3_)_2_ + 0.7NO + 1.3H_2_O + 0.05O_2_

3 UO_2_ + 4HNO_3_ → UO_2_(NO_3_)_2_ + 2NO_2_ + 2H_2_O

The highly oxidizing concentrated nitric acid environment favours NO_*x*_ species with N in the +4 oxidation state; for example, the oxidation of gaseous NO to NO_2_ by contact with concentrated aqueous HNO_3_ solutions shifts eqn (4)–(6) in favour of the products:

4 2NO(g) + O_2_(g) → 2NO_2_(g)

5 2NO_2_(g) ↔ N_2_O_4_(g)

6 2N_2_O_3_(l) ↔ 2NO(l) + N_2_O_4_(l)

Absorbed N_2_O_4_ can hydrolyse to nitrous and nitric acids *via*eqn (7); however, the extent of this reaction depends strongly on nitric-acid concentration and water activity. In water or dilute nitric acid, hydrolysis is important, whereas in concentrated nitric acid, a larger fraction of the absorbed N_2_O_4_ may remain in molecular form. Thus, N_2_O_4_ should be described as physically absorbed or dissolved in concentrated nitric acid.^37–39^

7 N_2_O_4_(l) + H_2_O(l) ↔ HNO_2_(l) + HNO_3_(l)

Nitrous acid also participates in coupled association and decomposition reactions. In the case of UNF processing, both desorption and absorption pathways are important. For example, the combination of eqn (6)–(8) yield the Abel-Schmid expression for aqueous HNO_2_ decomposition (eqn (9)).^37–39^

8 N_2_O_3_(l) + H_2_O(l) ↔ 2HNO_2_(l)

9 3HNO_2_(l) ↔ HNO_3_(l) + H_2_O(l) + 2NO(l)

Combining the principal gas-phase and liquid-phase reactions gives the commonly cited overall absorption stoichiometry, eqn (10):

10 3NO_2_(g) + H_2_O ↔ 2HNO_3_(l) + NO(g)

This equation indicates that removing three moles of NO_2_ from the gas phase produces one mole of gaseous NO, which is typically true for absorption with the low liquid–gas ratios that are commonly encountered in plate scrubbers. However, previous studies summarized by Counce and Perona^40^ found that the molar ratio of the production of gaseous NO to the NO_2_ absorbed only reached 1 : 3 under a semi-steady state HNO_2_ concentration, and was less prior to reaching steady-state. The reaction depended on the decomposition of aqueous HNO_2_ which, due to kinetic and mass transfer limitations, required a substantial HNO_2_ concentration as a driving force.^41–43^ This is important for scrubber design because the ratio of NO equivalents produced to NO_2_ equivalents absorbed may be substantially lower than the theoretical 1 : 3 ratio in the absence of the necessary concentration-based driving force during scrubbing. Given that NO_2_ is easier to scrub than NO, efforts to maximize the NO_2_/NO ratio and reduce the conversion of NO_2_ to NO during the treatment process should be emphasized. Here, more highly concentrated nitric acid us seen to promote NO_2_ formation by two means: (i) favouring NO_*x*_ with N in the +IV oxidation state (*i.e.*, by shifting eqn (4)–(6) to the right), and (ii) reducing the conversion of NO_2_ to NO by enhancing the nitrous acid concentration, eqn (7) and (8).

The scheme of flux equations for NO_*x*_ absorption into water or dilute HNO_3_ solutions is illustrated in Fig. 2. Gas-phase shows the oxidation of NO occurs with N_2_O_4_ and N_2_O_3_ in equilibrium with their respective reactant species, while the liquid N_2_O_4_ and N_2_O_3_ are shown reacting irreversibly with water in the liquid film (as is usually the case in packed towers with water or dilute aqueous HNO_3_ solutions as the absorbing solution). The reaction between NO_2_ and water may be neglected in most cases because it usually occurs in the bulk phase and is extremely slow. Note that the scheme of equations shown in the figure does not include gas-phase oxidation of NO by HNO_3_, as this reaction is not well understood, and the reader is referred to additional literature for an in-depth discussion.^44,45^

**Fig. 2:**
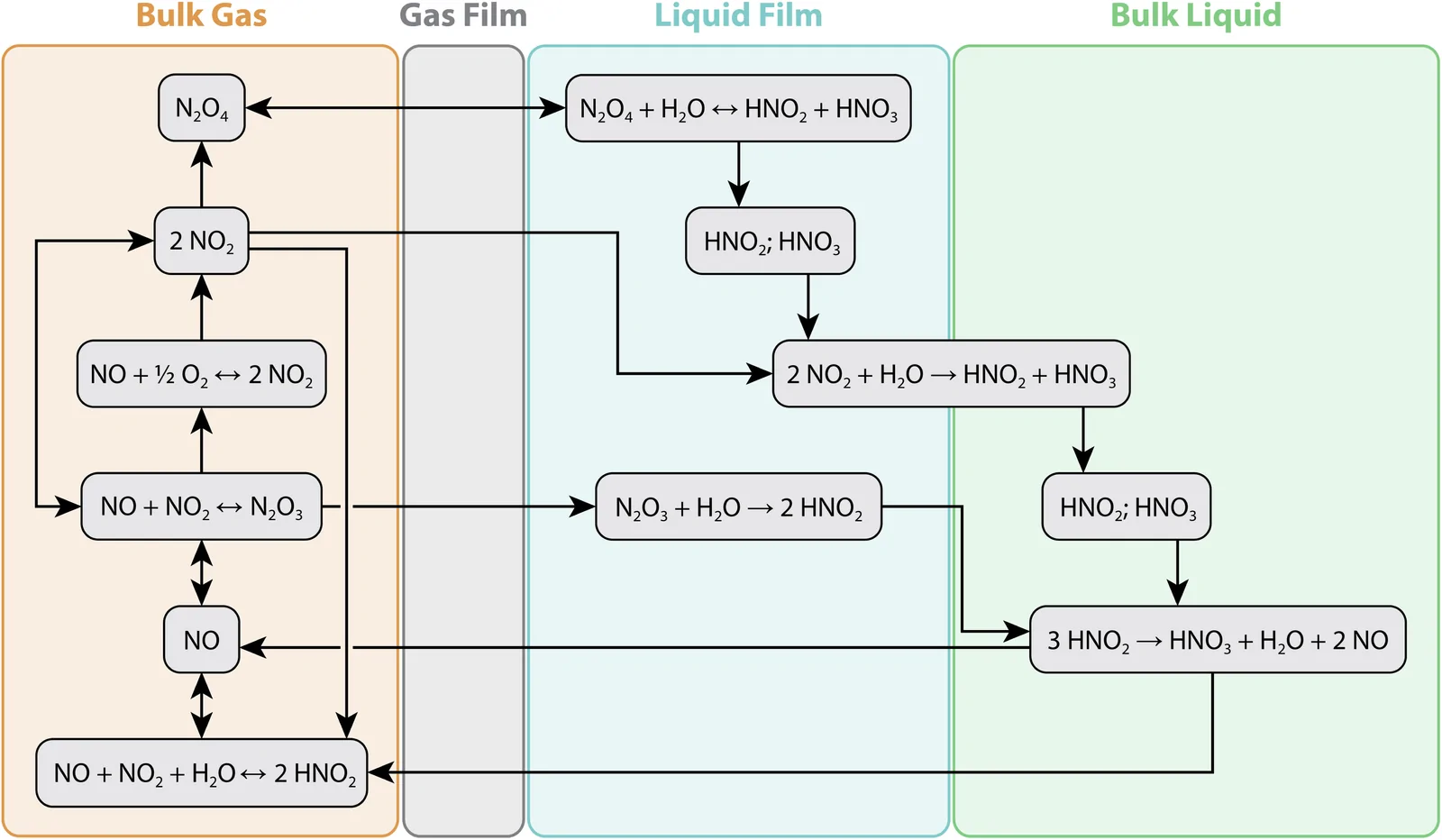
Flux equations for NO_*x*_ absorption into water or dilute HNO_3_ solutions. Reproduced with permission from Counce *et al.*^40^

NO_*x*_ absorption into concentrated HNO_3_ solutions is usually modelled empirically, based on a plate efficiency concept. To help bridge the gap between the mechanistically based models for absorption into dilute HNO_3_ and for absorption into concentrated HNO_3_, the enhancement factor (*Ek*_L_) may be used to determine nitric acid concentration for N_2_O_4_ absorption;^46^ the species N_2_O_3_ does not have a known similar parameter, but the variation may be similar to that of N_2_O_4_.

### 3.2 Physical properties of NO_*x*_ and other relevant compounds

The point of this section is to provide some order-of-magnitude differences in the properties of NO and NO_2_ against other target analytes present in the relevant streams (*e.g.*, inorganic iodine, organic iodine species). The chemical and thermal properties of NO and NO_2_ are very different, namely the melting temperatures (*T*_m_), boiling temperatures (*T*_b_), and densities (*ρ*). While NO has very low *T*_m_ and *T*_b_, NO_2_ is a liquid near room temperature. The density of the NO is 40% lower than NO_2_. A summary of the chemistry and properties of the NO_*x*_ constituents is provided in Table 2. These values are shown in Fig. 3(a) in comparison to the expected inorganic and organic iodine species that have been documented in representative off-gas streams.^47^ Based on Fig. 3(a), it is possible that these compounds could potentially be separated by employing their differences in physical properties (*e.g.*, fractional distillation). Compounds with similar *T*_m_ could be separated through differences in densities (Fig. 3(b)) such as NO_*x*_ and alkyl iodide compounds. In general, fractional distillation would not be the preferred method for separating out NO due to its very low boiling point (it would require cryogenic separation or conversion to NO_2_) but NO_2_/N_2_O_4_ could be captured this way. While the organic iodine compounds all have boiling points above room temperature, it is unlikely that fractional distillation could be used to capture these species.

**Table 2:** Summary of properties of NO_*x*_ gases (NO and NO_2_)

General name	Nitrogen(ii) oxide (nitric oxide)	Nitrogen(iv) oxide
IUPAC name	Nitrogen monoxide	Nitrogen dioxide
Chemical formula	NO	NO_2_
Nitrogen oxidation state	+2	+4
Appearance	Colorless	Brownish red gas
Odor	Slightly sharp	Pungent, irritating
Nature of oxide	Neutral	Acidic
Density (*ρ*)	1.3402 g L^−1^	1.880 g L^−1^
Melting temperature (*T*_m_)	109 K (−164 °C)	263.8 K (−9.3 °C)
Boiling temperature (*T*_b_)	121 K (−152 °C)	294.3 K (21.15 °C)
Hazards	Very toxic, corrosive, oxidizer	Highly toxic, oxidizer
Water solubility	Slightly soluble	Reacts with water
Reaction with water	No significant acid formation	Forms HNO_2_ and HNO_3_
Soluble within these solvents	HNO_3_, benzene, CCl_4_, gasoline, toluene	HNO_3_, CCl_4_, chloroform
Redox behavior	Oxidizing or reducing agent	Strong oxidizing agent
Magnetism	Paramagnetic	Paramagnetic

**Fig. 3:**
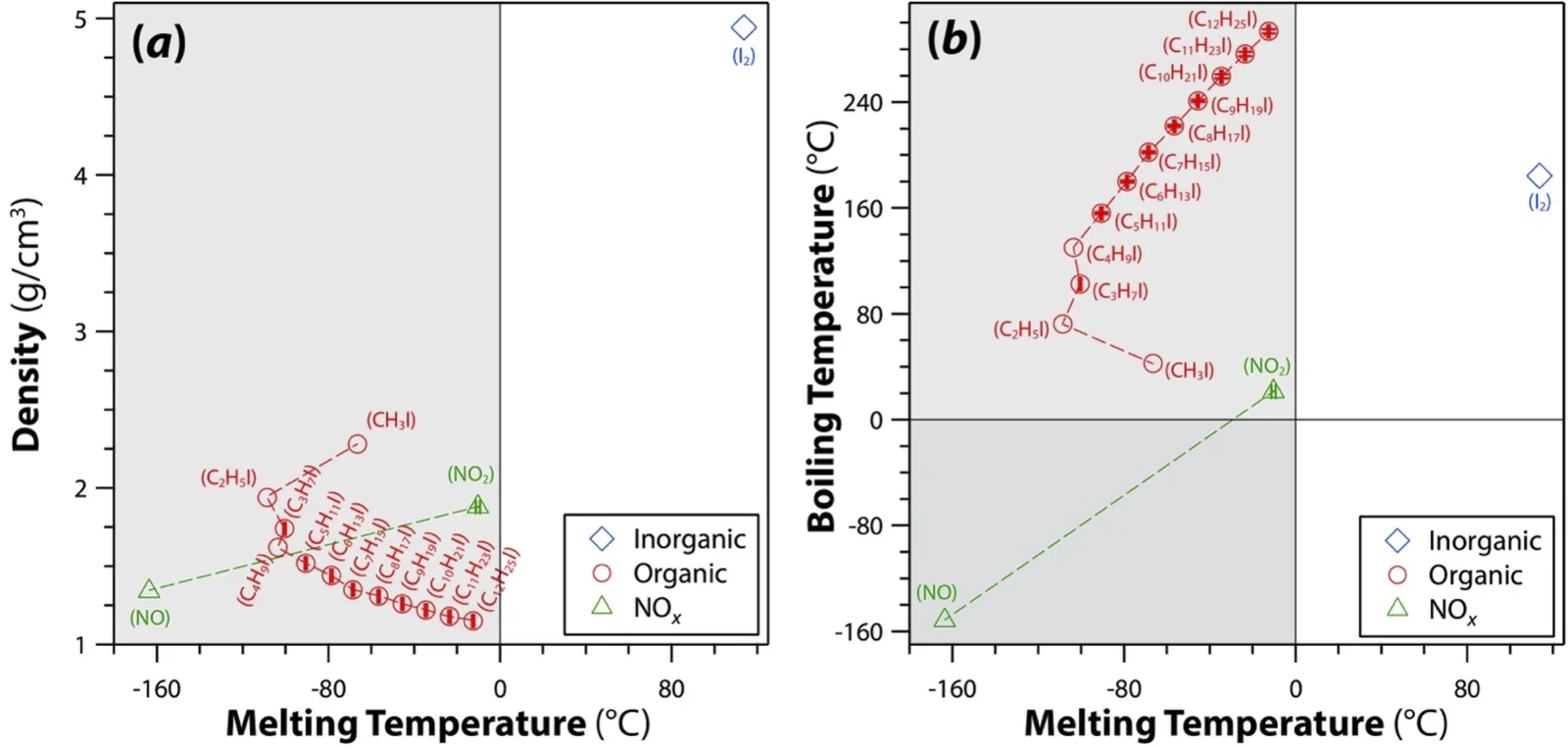
(a) Summary of densities *versus* melting temperatures for inorganic iodine [I_2_(g)], potential organic (alkyl) iodides [*e.g.*, CH_3_(CH_2_)_*x*_I, *x* = 0–11], which are estimated, as well as NO_*x*_ (*i.e.*, NO, NO_2_). (b) Boiling temperatures *vs.* melting temperatures for the same compounds. Lines are drawing between the common compound sets and temperature ranges of <0 °C are shaded.

### 3.3 NO_*x*_ rates and quantities

During UNF aqueous reprocessing by PUREX, the release quantities and rates of NO_*x*_ (as combination of NO and NO_2_) depend on a variety of unit operation parameters (temperature, pressure, dissolver liquid composition, *etc.*) as well as facility or plant-level considerations (throughput or batch size). Calculations of NO_*x*_ release rates on a per metric tonnes of heavy metal (MTHM) basis may proceed with these pathways in mind. As such, a representative facility with the characteristics listed in Tables S3 and S4 (SI) may give an indication of quantities to be expected. The forgoing calculation is predicated on an initial concentration of 8 M HNO_3_. The influence of HNO_3_ concentration and NO_*x*_ evolution are related. The reasoning behind this value is that the dissolution process is not an end unto itself, but a means of preparing the UNF for the solvent extraction process. The phase equilibria governing the distribution of uranium between the aqueous and organic phases is a strong function of HNO_3_. It is, therefore, ideal for the dissolution process to conclude with a final HNO_3_ concentration of ≥ 3 M. This permits minimal intervention at the feed adjustment stage. Quantities in this estimation were set such that the terminal concentration of HNO_3_ was ≈ 4 M. On the other hand, the overall rate of dissolution must be maintained such that the reaction does not slow down to a rate that impedes recycling facility throughput. An HNO_3_ stoichiometric excess of 100% was therefore assumed. Table 3 shows the resulting quantities of products with respect to the assumed reactant quantities. The resulting NO_*x*_ quantities in Table 3 were then calculated based on consensus values reported by Marc and coworkers.^36^

**Table 3:** Relationship between nitric acid concentration and normalized NO_*x*_ generation. Adapted from work compiled by Marc and coworkers^36^

[HNO_3_] (mol L^−1^)	Ratio (mol mol^−1^)	
	NO/UO_2_	NO_2_/UO_2_
3.4	0.64	0.07
4.5	0.62	0.08
6.7	0.61	0.13
8.1	0.54	0.43
12.5	0.36	1.06

Table 3 represents an estimate of source terms for both NO and NO_2_ normalized on a “per uranium input” basis. This enables extrapolations of NO_*x*_ quantities to larger facilities. Additionally, as the release quantity and speciation are strong functions of the particular flow sheet's nitric acid concentration, the off-gas handling system design requirements are tied to these processing variables. For an assumed processing facility operating at 200 MT per year at 200 operating days (1 MT per day) the following daily quantities are expected (Table 4):

**Table 4:** Calculation for daily release of NO_*x*_ for a 1 MTU per day throughput. Assumed HNO_3_ concentration and NO_*x*_ partitioning is based on the 8.1 mol L^−1^ value in Table 3

1 MT U per day	NO	NO_2_
Moles per day	2268.91	1806.72
Mass (kg per day)	68.07	54.20
Sum (kg per day)	122.27	

This total NO_*x*_ quantity (122.27 kg NO_*x*_ per MTU) is in line with reports from Vienna *et al.*^48^ (114.1 kg NO_*x*_ per MTU), who also puts it in perspective relative to other constituents of the dissolver off gas. Table 5 below shows a representation of the data presented by Vienna *et al.*^48^ As can be readily seen, the combined NO_*x*_ emissions are significantly larger than the other constituents of interest and must be dealt with according to its preponderance in the overall gas stream. This data represents used LWR fuel that was 5% enriched, Zircaloy-4 clad, irradiated for 50 gigawatt day (GWd per tU) in a pressurized water reactor (PWR) and cooled for 5 years before reprocessing.

**Table 5:** Mass quantities and fractions of constituents of interest in dissolver off-gas streams. Table adapted from Vienna *et al.*^48^

Component	Quantity (kg per MtU)	Mass fraction
^14^C	3.00 × 10^−4^	2.44 × 10^−6^
^3^H	5.80 × 10^−11^	4.72 × 10^−13^
Total iodine	0.33	2.69 × 10^−3^
Total krypton	0.53	4.32 × 10^−3^
Total xenon	7.8	6.35 × 10^−2^
Total NO_*x*_	114.1	9.29 × 10^−1^

## 4 Voloxidation (dry head-end pretreatment)

Voloxidation (or volox) is a process by which used oxide fuel is treated during head-end processing at elevated temperatures in an oxidizing environment to oxidize UO_2_ to U_3_O_8_ or UO_3_.^16,49,50^ Voloxidation results in volume expansion of the fuel where this volume expansion results in the release of trapped fission gases (*e.g.*, ^3^H, ^14^C, ^85^Kr, ^129^I). Pulverizing the fuel aids in subsequent dissolution and significantly increases the specific surface area, as well as separating the fuel and cladding.^16,51,52^ Advanced voloxidation (advanced volox) is referred to as heterogeneous NO_2_-mediated oxidation of uranium. In advanced voloxidation, NO_2_ is the oxidizing gas. A comparison of voloxidation and advanced voloxidation is provided in Table 6, including the release of volatile fission gases in each scenario. Thus, one major source of NO_*x*_ generation in reprocessing occurs with advanced voloxidation.

**Table 6:** Comparison of voloxidation and advanced voloxidation^2,5,49^

Parameter	Voloxidation (volox)	Advanced voloxidation (advanced volox)
Temperature	400–600 °C	200–500 °C
Atmosphere	Air, pure O_2_(g), or pure O_3_(g)	NO_2_(g) (or NO_*x*_ mixtures)
Radionuclides (and other species) released	^3^H (>99%), partial amounts of ^14^C, ^85^Kr, Xe	^3^H, ^14^C, ^85^Kr, ^129^I (all 100%); Cs (98%), Ru (97.6%), Rb (96%), Tc (92.3%), Rh (82.7), Te (53.3%), Mo (61.7%)

## 5 NO_*x*_-driven iodine chemistry and sorbent degradation

Iodine speciation in aqueous reprocessing is controlled by fuel chemistry, HNO_3_ dissolution, NO_*x*_/HNO_2_ redox chemistry, and subsequent contact with solvent and waste-treatment streams. Although iodine is often treated as a single volatile fission product, it can occur as metal iodides, molecular iodine, iodate, organic iodides, colloidal iodide phases, particulate iodine, or silver-bound iodide/iodate on capture media. Operating plants and plant-scale tests found that the majority (94–99%) of ^129^I is released into the DOG with the remainder reporting to other operations in the separation vessel off-gas (VOG).^47,53^

### 5.1 NO_*x*_ effects on iodine sorbent beds

Despite the need for radionuclide separation from UNF reprocessing off-gas, the high NO_*x*_ gas concentrations in the off-gas stream remains a considerable challenge to radionuclide capture.^54^ The high reactivity of NO and NO_2_ can introduce competing reaction pathways that diminish sorbent capacity or generate undesirable byproducts. The magnitude of this effect depends on NO_*x*_ composition, concentration, exposure time, temperature, humidity, test configuration (flowing stream *versus* sealed static vessel) and sorbent chemistry and is not necessarily linear with concentration or duration. In some silver-based sorbents, a rapid initial loss in iodine capacity is observed, followed by comparatively little additional degradation with continued exposure.^17–21^ A prime example of this can be seen in the context of radioiodine capture. Traditionally, silver-based adsorbents (*e.g.*, silver-exchanged mordenite, AgZ, or Ag^0^Z if reduced; silver nanoparticle-functionalized aerogels, Ag-aerogel) have been regarded as highly promising materials for radioiodine capture due to their strong chemisorption of iodine in the form of a highly stable silver iodide.^55,56^ NO_*x*_ exposure through aging experiments has been shown to dramatically reduce the iodine capacities of these silver-based adsorbents (*i.e.*, more than 90% loss in capacity under flowing NO_2_) as shown by empirical data collected in several experimental studies, which are summarized in Table 7.^57–60^

**Table 7:** Iodine loading capacity of reduced silver mordenite (Ag^0^Z) and silver-functionalized silica aerogel (Ag-aerogel) before (*q*_0_) and after (*q*_aged_) aging at 150 °C. Rows are grouped by aging atmosphere. Each capacity loss is calculated against the unaged baseline of the same study, 100 × (1 − *q*_aged_/*q*_0_). Values are directly comparable only within a study; across studies they indicate trends only, because sorbent batches, silver content, reduction conditions, aging mode (flowing *versus* sealed static), humidity, iodine-loading conditions, and quantification method (TGA mass gain *versus* NAA) all differ. Rows marked b share matched aging gases and one iodine-loading protocol and can be compared across sorbents^a^

Sorbent	Material and pretreatment	Aging atmosphere and mode	Aging time	*q*_0_ (mg I g^−1^)	q_aged_ (mg I g^−1^)	Capacity loss (%)	Iodine-loading and quantification method	Source
**Dry air**								
Ag^0^Z	IONEX Ag-900, 9.5 wt% Ag; 4% H_2_/N_2_, ∼200 °C	Flowing dry air (dew point ≈ −55 °C), 9.6 m min^−1^	6 months	≈90^c^	≈54	40	Single-pellet TGA; 250 mg I_2_ m^−3^; mass gain	Jubin *et al.* (2012)^61^
Ag^0^Z	IONEX Ag-900 (Ag content not stated); 4% H_2_/Ar, 400 °C, 24 h	Flowing dry air (dew point < −70 °C)	1 week	123^d^	105	15	Microbalance (screen pan); 50 ppmv I_2_ in dry air, 150 °C; to constant mass	Choi *et al.*^b^(2021)^62^
			1 month		80	35		
			2 months		≈82	≈33		
			4 months		≈81	≈35		
			6 months		≈75	≈39		
Ag-aerogel	Ag^0^-functionalized silica aerogel (PNNL)	Flowing dry air^e^	6 months	408	320	22	TGA loading; 150 °C; 11.85 L min^−1^ air stream 56 ppm of iodine; physisorbed iodine removed through 24 h air purge^e^	Matyáš *et al.* (2018)^58^
Ag-aerogel	Ag^0^-functionalized silica aerogel (PNNL); 2.7% H_2_/Ar, 165 °C, 2 h; 1 h N_2_, 150 °C before aging	Flowing dry air (dew point < −70 °C)	1 month	364^f^	321	12	Microbalance (screen pan); 50 ppmv I_2_ in dry air, 150 °C; to constant mass	Choi *et al.*^b^(2021)^63^
			2 months		≈321	≈12		
			4 months		≈298	≈18		
			6 months		295	19		
**Humid air**								
Ag^0^Z	IONEX Ag-900, 9.5 wt% Ag; 4% H_2_/N_2_, ∼200 °C	Flowing humid air (dew point +15 °C), 9.6 m min^−1^	1 month	>110^c^	≈50	≈55	Single-pellet TGA; 200 mg I_2_ m^−3^, 9.7 m min^−1^, 150 °C; mass gain	Jubin *et al.* (2012);^61^ Bruffey *et al.* (2015)^18^
			4 months		—	60^g^		
Ag^0^Z	IONEX Ag-900 (Ag content not stated); 4% H_2_/Ar, 400 °C, 24 h	Flowing humid air (dew point −15 °C)	1 week	123^d^	≈61	≈50	Microbalance (screen pan); 50 ppmv I_2_ in dry air, 150 °C; to constant mass	Choi *et al.*^b^(2021)^62^
			1 month		62	49		
			2 months		≈56	≈55		
			4 months		≈53	≈57		
			6 months		≈51	≈59		
Ag-aerogel	Ag^0^-functionalized silica aerogel (PNNL)	Flowing humid air^e^	6 months	307	239	22	TGA loading; 150 °C; 11.85 L min^−1^ air stream 56 ppm of iodine; physisorbed iodine removed through 24 h air purge^e^	Matyáš *et al.* (2018)^58^
Ag-aerogel	Ag^0^-functionalized silica aerogel (PNNL); 2.7% H_2_/Ar, 165 °C, 2 h; 1 h N_2_, 150 °C before aging	Flowing humid air (dew point −15 °C)	1 month	364^f^	298	18	Microbalance (screen pan); 50 ppmv I_2_ in dry air, 150 °C; to constant mass	Choi *et al.*^b^(2021)^63^
			2 months		≈270	≈26		
			4 months		≈257	≈29		
			6 months		≈267	≈27		
**NO**_**2**_ **, static (sealed vessel; finite NO**_**2**_ **inventory)**								
Ag^0^Z	IONEX Ag-900	Static ∼2% NO_2_ in air, sealed	2 months	59	41	30^h^	TGA loading; 150 °C; 11.85 L min^−1^ air stream 56 ppm of iodine; physisorbed iodine removed through 24 h air purge^e^	Matyáš *et al.* (2018)^58^
Ag-aerogel	Ag^0^-functionalized silica aerogel (PNNL); ∼35 wt% Ag (NAA); 10 g per holder	Static 2% NO_2_, 150 °C, sealed 6-in stainless-steel holders	1 month	331	339	None^i^	TGA, 56 ppm I_2_, 10.3 m min^−1^ (loading temperature not stated); run until no further mass gain, then 24 h dry-air purge; q values from TGA, confirmed by NAA	Patton *et al.* (2014b)^64^
			2 months		347	None^i^		
			4 months		283	15 (TGA); 31 (NAA)^i^		
**NO**_**2**_ **, flowing**								
Ag^0^Z	IONEX Ag-900, 11.9 wt% Ag; 4% H_2_/Ar, 400 °C, 24 h	Flowing 2% NO_2_ in dry air	12 min	123	51	59	≈0.2 g on microbalance screen; 50 ppmv I_2_ in dry air, 150 °C; run to constant mass, then clean-air purge	Wiechert *et al.*^b^(2020)^60^
			5 h		14	89		
			1 month		9.8	92		
			2 months		6.6	95		
			4 months		3.0	98		
			6 months		3.5	97		
Ag-aerogel	Ag^0^-functionalized silica aerogel (PNNL); 2.7% H_2_/Ar, 165 °C, 2 h; 1 h N_2_, 150 °C before aging	Flowing 2% NO_2_ in dry air	1 month	364^f^	≈50	≈86	Microbalance (screen pan); 50 ppmv I_2_ in dry air, 150 °C; to constant mass	Choi *et al.*^b^(2021)^63^
			2 months		≈42	≈88		
			4 months		<20	>94		
Ag-aerogel	Ag^0^-functionalized silica aerogel (PNNL); ∼35.5 wt% Ag; 100 mg; 1 h N_2_, 150 °C before aging; ground to powder after aging	Flowing 2% NO_2_ in dry air, 150 °C, 500 mL min^−1^	1 day	375	≈200^j^	≈47	Microbalance (0.1 µg); 50 ppmv I_2_ in dry air, 150 °C, same flow rate as aging; to equilibrium mass	Shen *et al.*^b^(2022)^59^
			1 week		≈128^j^	≈66		
			2 weeks		≈94^j^	≈75		
			1 month		42^j^	89		
**NO, flowing**								
Ag^0^Z	IONEX Ag-900, 9.5 wt% Ag; 4% H_2_/N_2_, 270 °C, 10 days	Flowing 1% NO in dry air, 3.5 m min^−1^ (∼10% of NO estimated to convert to NO_2_)	1 week	≈90	≈20	78	TGA, 50 ppm I_2_, 10 m min^−1^, to no further mass gain, then 24 h dry-air purge; mass gain	Patton *et al.* (2014)^65^
			1 month		≈20	78		
Ag^0^Z	Same samples as row above	Same as row above	1 week	77.5	35.5	54	NAA of the TGA-loaded samples^k^	Patton *et al.* (2014)^65^
			1 month		29.3	62		
Ag^0^Z	IONEX Ag-900, 11.9 wt% Ag; H_2_ reduction, 230 °C	Flowing 1% NO in dry air; 34 g deep bed, 3.5 m min^−1^, subsampled	4 weeks	97	27	72	TGA loading; final loading by NAA	Bruffey *et al.* (2015)^66^
			8 weeks		15	85		
Ag^0^Z	IONEX Ag-900, 11.9 wt% Ag; 4% H_2_/Ar, 400 °C, 24 h	Flowing 1% NO in N_2_ (no O_2_)	1 day	123	75	39	≈0.2 g on microbalance screen; 50 ppmv I_2_ in dry air, 150 °C; run to constant mass, then clean-air purge	Wiechert *et al.*^b^(2020)^60^
			1 week		50	60		
			1 month		14	89		
			2 months		16	87		
			4 months		13	90		
			6 months		4.7^l^	96		
Ag-aerogel	Ag^0^-functionalized silica aerogel (PNNL)	Flowing 1% NO in dry air (dew point < −70 °C); 20.4 g deep bed, 3.5 m min^−1^, subsampled	1 month	290	220	24	TGA, 50 ppm I_2_ in dry air, 10 m min^−1^, 150 °C; final gravimetric loading	Bruffey *et al.* (2015)^18^
			2 months		165	43^m^		
Ag-aerogel	Ag^0^-functionalized silica aerogel (PNNL); 2.7% H_2_/Ar, 165 °C, 2 h; 1 h N_2_, 150 °C before aging	Flowing 1% NO in N_2_ (no O_2_)	1 month	364^f^	281	23	Microbalance (screen pan); 50 ppmv I_2_ in dry air, 150 °C; to constant mass	Choi *et al.*^b^(2021)^63^
			2 months		≈260	≈29		
			4 months		232	36		

a Abbreviations: TGA, thermogravimetric analysis; NAA, neutron activation analysis. Capacities reported in wt% were converted as 1 wt% = 10 mg I g^−1^ sorbent. — indicates not reported numerically in the source.

b Choi *et al.* (2021),^62,63^ Wiechert *et al.* (2020),^60^ and Shen *et al.* (2022)^59^ come from one research group, used matched aging gases (dry air, dew point < −70 °C; humid air, dew point −15 °C; 1% NO in N_2_; 2% NO_2_ in dry air; all at 150 °C), and loaded iodine on a microbalance at 50 ppmv I_2_ and 150 °C. These rows are the most directly comparable between the two sorbents, although reduction conditions differ between Ag^0^Z and Ag^0^-aerogel.

c Jubin *et al.* (2012)^61^ report ∼9 wt% mass gain for unaged Ag^0^Z after ∼90 h (dry air, 250 mg I_2_ m^−3^) and a ∼40% reduction after 6 months of dry-air aging; the aged value is calculated from these. The same paper compares the humid-air 1 month value (∼5 wt%) with “over 11 wt%” for freshly reduced Ag^0^Z.

d Choi *et al.* (2021)^62^ state 12.3 wt% (unaged), 10.5 wt% (1 week) and 8.0 wt% (1 month) for dry air and a 49.4% loss after 1 month in humid air; all other values are read from their Fig. 3 (reading uncertainty roughly ±0.2 wt%). The ∼40% (dry air) and ∼50% (humid air) losses over 6 months quoted in that paper refer to earlier ORNL studies, not to its own measurements.

e TGA was configured from a VWR model 1330GM and a Mettler–Toledo XS603S weighing pan. Samples were dried under N_2_ at 150 °C.

f Choi *et al.* (2021).^63^ Stated in the text: 36.38 wt% (unaged); dry air 32.11 (1 month) and 29.45 (6 months); humid air 29.77 (1 month); 1% NO/N_2_ 28.05 (1 month) and 23.16 (4 months); 2% NO_2_ ∼5 wt% (1 month) and less than 2 wt% (4 months). All other values are read from their Fig. 3 (roughly ±0.5 wt%). Losses are calculated against 36.38 wt%. The 6 months humid-air value in Fig. 3 (≈26.7 wt%) is a slight rise over the 4 months value and gives a larger loss than the 22% the authors cite from earlier studies.

g From Bruffey *et al.* (2015),^66^Table 1 (4 months). Jubin *et al.* (2012)^61^ state that the 6 months humid-air sample was not loaded; Matyáš *et al.* (2018)^58^ list the same 60% loss (97 → 39 mg I g^−1^) as a 6 months result.

h Bruffey *et al.* (2015, Table 1)^66^ restate the 30% loss after 2 months of static 2% NO_2_ and attribute it to an August 2013 ORNL letter report (ORNL/LTR-2013/351). Jordan and Bruffey (2016)^67^ cite a 25–50% loss for 1–2 months of static 2% NO_2_ aging. The unaged capacity (59 mg I g^−1^) is lower than for the other Ag^0^Z samples in this table.

i TGA: 33.1, 33.9, 34.7, and 28.3 wt%; NAA: 33.5, 30.9, 34.3, and 23.2 wt% at 0, 1, 2, and 4 months. The source reports very limited loss in the first 2 months. Bruffey *et al.* (2015)^18^ and Jordan and Bruffey (2016)^67^ assign this 15% loss to 2 months; the originating report places it at 4 months.

j Shen *et al.* (2022):^59^*q*_0_ = 37.5 wt% and the 1 month value (4.2 wt%, 11% of original) are stated in the text. The 1 day to 2 weeks values are read from Fig. 2 (graphical, uncertainty roughly ±1 wt%). Fig. 2 shows ≈5.6 wt% at 28 days (≈85% loss). The text assigns 9.5 wt% (25% of original) to 7 days, whereas Fig. 2 shows ≈12.8 wt% at 7 days and ≈9.4 wt% at 14 days; Fig. 2 is used here. Samples were ground to powder after aging, unlike the granule/pellet tests above.

k NAA reports iodine as a fraction *w* of the loaded sample mass; converted here as *q* = *w*/(1 − *w*): 7.19, 3.43, and 2.85 wt% correspond to 77.5, 35.5, and 29.3 mg I g^−1^.

l Table 1 of Wiechert *et al.* (2020)^60^ gives 0.47 wt%; the text gives 0.45 wt%.

m Fines (∼30 wt% of the bed after the 1 week sampling) were removed and not returned for further aging, so the 2 months value describes the remaining granular material only.

In one study of aging Ag^0^Z in flowing NO_2_/air and NO/N_2_, capacity loss was attributed to oxidation of the Ag^0^ nanoparticles into forms unavailable for iodine adsorption. Nitrates were identified as the primary oxidation product, present as molecular surface species rather than bulk AgNO_3_, and these nitrates accounted for most of the iodine still adsorbed by aged materials.^54^ For Ag-aerogel, in which most of the silver is bound to the thiol groups tethered to the aerogel, NO_*x*_ oxidizes the sulfur to sulfate, and the resulting Ag_2_SO_4_ silver sulfate aggregates into particles that no longer adsorb iodine.^58,59,68^ Under NO and air, the smaller losses have been attributed to the formation of silver sulfate, and under NO by growth of the silver nanoparticles^58^ or to conversion of Ag^0^ to Ag_2_S;^63^ however, the studies do not agree on the sulfur product. Therefore, while mechanisms for iodine capture capacity loss have been proposed for both Ag^0^Z and Ag-aerogel, the oxidation pathways and final products remain unconfirmed.

Due in part to the effect of NO_*x*_ on silver adsorbent capacity as well as concerns surrounding adsorbent cost and toxicity, there has been a recent effort to explore alternative metal adsorbents for radioiodine capture.^25,69–71^ While some materials boast a greater resistance to NO_*x*_ than their silver counterparts, they are nonetheless still significantly impacted by NO_*x*_ in the off-gas stream.^3,19,72^

Several issues are associated with utilizing silver as an active getter in solid sorbent media that include, at the forefront, the rising cost of silver but also the environmental toxicity of silver. In the U.S., the disposal of silver is controlled by Environmental Protection Agency under the Resource Conservation and Recovery Act (RCRA) through 40 CFR 261 along with seven other elements (*i.e.*, As, Ba, Cd, Cr, Hg, Pb, Se). In response to this, other active getters are being explored for iodine capture, including primarly Bi (Bi^0^, Bi^3+^) and Cu (Cu^0^, Cu^+^), which are much less noble than Ag. However, very few studies have been conducted to assess these materials in NO_*x*_ environments. The preliminary work^16,25,47,50,73^ in this area shows that, while NO_*x*_ does degrade performance of Bi-functionalized and Cu-functionalized sorbents, the degradation is in-line with values associated with Ag-sorbents. However, more work is needed to assess the degradation effects of NO_*x*_ exposure these types of replacement options for Ag-based sorbents.

### 5.2 NO_*x*_ effects on iodine speciation

While multiple options exist for ^129^I capture (*e.g.* solid sorbents and liquid scrubbers), these methods are highly dependent upon iodine form and speciation (*e.g.*, inorganic I_2_, CsI, alkyl iodides, including CH_3_(CH_2_)_*x*_I where *x* = 0–11) and are affected by other contaminants in the off-gas.^54,74^ If the capture methods are incompatible with the speciation of the released iodine, system performance will be poor.^5^ Examples of performance degradation within iodine solid sorbents (*e.g.*, AgZ, Ag-aerogel) in the presence of NO_*x*_ were discussed above (Table 7) and show how much these gases can affect the iodine binding mechanisms (*e.g.*, Ag^0^ or Ag^+^ conversion to AgNO_3_).

In oxide fuel, iodine is expected to be stabilized primarily as iodide salts, especially CsI, because cesium is an abundant fission product and highly electropositive. In a study by Johnson *et al.*,^75^ they showed that NO_2_ exposure of CsI can result in the production of CsNO_3_ and I_2_, which simplifies downstream iodine capture. However, silver and palladium fission products will form low-solubility colloidal iodides, particularly AgI and PdI_2_,^76^ and persist as undissolved solids during downstream processing. Sakurai *et al.*^76^ determined that NO_2_ sparging in the dissolver can suppress the decomposition of AgI and PdI_2_ colloids whereas heating the dissolver solution in the absence of NO_2_ can assist with colloid decomposition (and adding IO_3_^−^ can accelerate decomposition). This is another example of how minimizing NO_2_ exposure to streams can improve and help optimize the distribution of iodine species present in these unit operations.

Generally, in conventional aqueous-based UNF reprocessing, formation of I_2_ is favoured during active dissolution because the environment promotes oxidation of iodide to I_2_ while also reducing iodate back to I_2_, thereby limiting significant iodate accumulation.^76^ Caustic scrubbers are effective for I_2_ but much less effective for organic iodides because these species have lower solubility and slower reaction rates.^4^ Therefore, a scrubber designed to capture NO_*x*_ may selectively remove I_2_ while allowing organic iodides to pass to downstream capture beds. In that case, the total iodine in the off gas exiting the scrubber will decrease, but the remaining iodine may be more difficult to capture. Adding a strongly oxidizing reagent to the caustic scrubber, such as sodium hypochlorite can oxidize captured iodine to non-volatile iodate or periodate forms, increasing liquid retention; however, the resulting iodine-bearing solution would require further treatment prior to disposal.^77,78^

Acidic NO_*x*_ absorption or nitric-acid recovery may instead allow iodine to remain volatile as I_2_, but iodine contamination of recycled acid becomes a potential concern. A process that removes I_2_ efficiently from the dissolver off-gas may still underperform at the plant scale if iodine is allowed to enter solvent extraction and later emerge as organic iodides in vessel off-gas or solvent-treatment off-gas. Another acidic scrubbing process for iodine is referred to as Iodox (hyperazeotropic, 20–23 M, HNO_3_) with DF > 10^4^. In this process, CH_3_I can be converted to I_2_, I_2_ can be converted to I^+^, and I^+^ can be converted to IO_3_^−^.^79,80^ Minimizing the NO_*x*_ concentrations in the off-gas streams could help reduce the oxidized iodine species (*e.g.*, IO_3_^−^), which are problematic to capture in downstream processes and have a more challenging route for waste form production and disposal. This is in part due to the lower iodine loading (*i.e.*, iodine is diluted by oxygen) for iodate (IO_3_^−^) *vs.* iodide (I^−^) or iodine (I_2_).

## 6 NO_*x*_ control technologies

Multiple technologies for NO_*x*_ abatement are discussed below, including wet scrubber systems, dry methods, such as solid sorbents, and selective catalytic reduction, as well as emerging technologies, such as membrane-based separations.

### 6.1 Wet NO_*x*_ control

Only low concentrations of NO_2_ are expected in the off-gas of conventional fuel reprocessing facilities. However, newer approaches to used fuel recycling that involve the direct injection of NO_2_ may result in high concentrations of NO, a byproduct of fuel oxidation, as well as unreacted NO_2_. Thus, this section on wet scrubbers focuses on the broad range of NO_*x*_ that could be of concern and the methods that could be used to remove high concentrations of NO_*x*_ following proven industrial processes. In general, the trapping of NO_*x*_ species requires shifting the chemical equilibrium between NO/NO_2_ towards oxidation and production of HNO_3_, Fig. 2, sometimes through the intermediate N_2_O_5_, as shown in eqn (11) and (12).

Across various industries, the presence of NO_*x*_ gases in process streams can present challenges in terms of material compatibility that make it difficult to certify systems to environmental and public safety regulations. A common solution found for various processes that may result in the production of NO_*x*_ is to pass the process or waste stream through a wet scrubber system. When considering wet (liquid)-scrubbing methods, two approaches are commonly employed: acidic scrubbing and caustic scrubbing. Wet scrubbing options for NO and/or NO_2_ that have been documented in the literature include solutions of NaOH,^81^ Ca(OH)_2_,^81^ H_2_O_2_ with HNO_3_,^82^ NaClO_2_ (sodium chlorite),^83^ and KMnO_4_ (potassium permanganate).^84^ The pollutants will either dissolve or react with the solution before the scrubbed gas is released, preventing downstream materials from being exposed to NO_*x*_. In acidic scrubbing, the off-gas stream passes through a condenser, which recovers water and nitric acid. The partially cooled gas is then directed through an acid absorption or nitric-acid recombination column, where NO_2_ is converted to HNO_3_. The recovered acid can then be recycled to the dissolution process. In caustic scrubbing, residual NO_*x*_ is absorbed into an alkaline solution, typically NaOH, and converted to sodium nitrite and sodium nitrate. A proposed example of a hybrid acidic-caustic scrubber system is provided in Fig. 4. In addition to utilizing these approaches, autotitration can be performed to automate these types of liquid-based neutralization reactions where, if the target pH changes outside of a specific threshold, additional titrant can be added to the scrubber container.

**Fig. 4:**
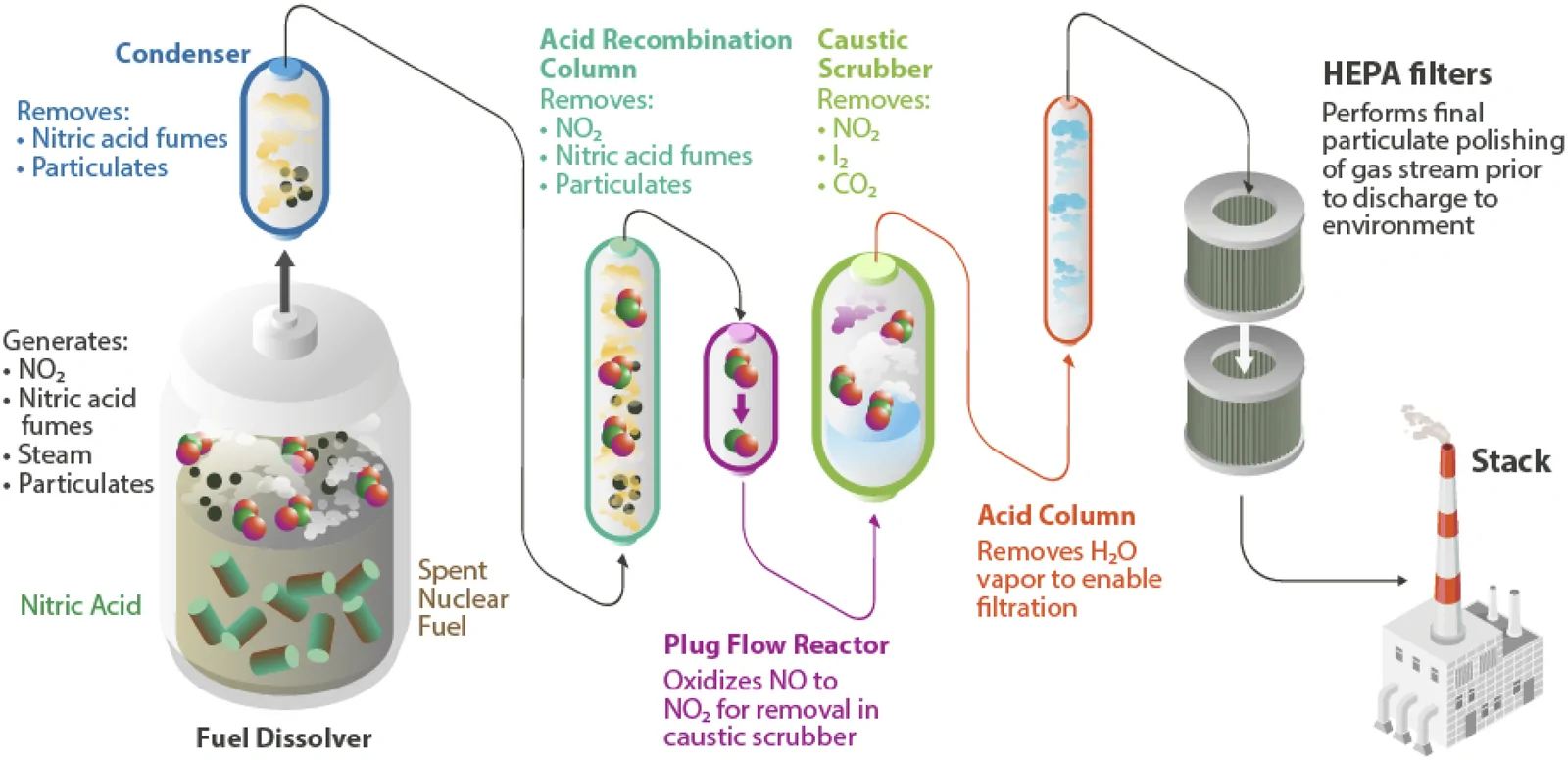
Hybrid acidic-caustic scrubber system diagram for removal of nitrogen oxides from aqueous nuclear fuel reprocessing off-gas.

Another example of wet scrubbing systems that can be used to capture NO_*x*_ from process streams includes the Low Temperature Oxidation (LoTOxTM) Process.^85^ This is a technology that oxidizes the NO_*x*_ at low temperatures (∼150 °C), converting it into soluble compounds that can be absorbed by the liquid solution in the scrubber. This is accomplished by injecting ozone into the gas flow, which will then react with the NO and NO_2_ vapor species to form soluble N_2_O_5_ as shown in eqn (11) and (12). The N_2_O_5_ will react rapidly with moisture in the gas stream to form HNO_3_, which can be collected and stored for disposal or for recycling. The rapid reaction rate of the ozone with NO_*x*_ is irreversible and allows for nearly complete removal of the NO_*x*_ from the gas stream. The challenge in applying these methods to a UNF off-gas stream is that NO_*x*_ gas will comingle with the many radioactive elements, such as ^14^C and ^129^I, released during processing. Thus, the complex mixture may not lend itself to a simple separation of NO_*x*_ without subsequent downstream process steps, required prior to disposal or recycle.

11 2NO_2_ + O_3_ → N_2_O_5_ + O_2_

12 2NO + O_3_ → N_2_O_5_

One last liquid scrubbing process that has been demonstrated to capture NO_*x*_ is acidic solution with hydrogen peroxide as an oxidizer (H_2_O_2_).^82,86^ Within a study by Ghriss *et al.*,^82^ where the acid was up to 8 M HNO_3_ along with 0–2 M H_2_O_2_, the general findings reported: (1) the efficiency of NO_*x*_ absorption in the scrubber was higher with a higher proportion of oxidized species in the NO_*x*_ mixture; (2) the addition of H_2_O_2_ to the HNO_3_ drastically increased NO_*x*_ absorption; (3) NO_*x*_ absorption increased with initial H_2_O_2_ and HNO_3_ concentrations; (4) without H_2_O_2_ present, NO_*x*_ abatement decreased when the concentration of HNO_3_ was increased; and (5) without H_2_O_2_ present, NO_*x*_ abatement decreased with recycle of the scrubbing liquid leading to NO_*x*_ desorption.

Demonstrated scrubber solutions for effective trapping a range of NO concentrations are shown in Table 8. For caustic wet scrubbing, Sodium chlorite (NaClO_2_) solutions with^83,87,88^ and without^83,89,90^ the addition of NaOH have been studied for NO capture and NO_2_ capture. The experimental conditions used for testing in some example studies are summarized in Table 8. NaClO_2_ is most effective at oxidizing NO to NO_2_ at lower pH values while the absorbing capacity for NO_2_ retention is highest at high pH values.^91,92^

**Table 8:** Summary of experimental conditions used in evaluating basic capture liquids

Stream description	Additive-1	Additive-2	*T* (°C)	pH	Reference
0.8–15 vol% NO	0.2–1.5 M NaClO_2_	0.05–0.5 M NaOH	25	—	Sada *et al.*^87^
0.5–7.5% NO	0.29–1.64 M NaClO_2_	0.015 M NaOH	25	—	Sada *et al.*^88^
200–1000 ppm NO	0.05–1.0 M NaClO_2_	—	20	9.2–9.9	Brogren *et al.*^89^
290 ppm NO	0.1–1.0 M NaClO_2_	—	20	8–11	Hsu *et al.*^90^
300–800 ppm NO	0–1.5 M NaClO_2_	0–0.3 M NaOH	50	—	Chu *et al.*^83^
250 ppm NO, 100 ppm SO_2_	0.2 M NaClO_2_	—	—	—	Pourmohammadbagher *et al.*^91^

### 6.2 Solid sorbents

Dry sorbent systems may offer operational advantages over wet scrubbing and can be implemented as fixed or replaceable packed beds or by dry sorbent injection.^19,82^ Unlike wet scrubbers, these systems generally do not require continuous circulation of an absorbing liquid and avoid scaling associated with crystallization from concentrated scrub solutions. However, they still require gas-handling and solids-management equipment, and their footprint and cost depend on the sorbent capacity, required residence time, pressure drop, replacement frequency, and whether regeneration is employed. Their performance is also sensitive to gas flow rate and composition because changes in space velocity, gas–solid contact time, humidity, and contaminant loading can affect removal efficiency and bed breakthrough. Dry systems can accommodate variable operation when appropriately sized and controlled, but they are not insensitive to flow fluctuations.^93^

The removal of NO_*x*_ gasses using solid sorbents can generally be categorized into three groups: temporary NO_*x*_ storage on porous solids (activated carbon, zeolites),^94^ catalytic transformation (SCR/NSR catalysts),^94^ and alkaline neutralization.^95^ Solid sorbent selection for a given process not only depends on its mechanism, but also gas composition, humidity and temperature.

#### 6.2.1 Temporary NO_*x*_ storage

Temporary NO_*x*_ storage relies on reversible adsorption or surface reaction of NO_*x*_ species on porous or chemically active solids. The objective is to retain NO_*x*_ on the solid during periods of operation and regenerate and/or subsequently process the captured species. Activated carbon^96^ and zeolites are two examples of sorbent materials that have been tailored for a variety of applications. Activated carbon impregnated materials prepared with potassium hydroxide^9^ or phosphoric acid^7^ have demonstrated enhanced performance for NO_*x*_ capture. Transition-metal-exchanged zeolites, such as Cu-loaded^97^ or Fe-loaded^98^ ZSM-5, temporarily store NO_*x*_ species by forming surface-bound nitrosyl, but may also form irreversible nitrate or nitrite complexes. The combination of high specific surface areas provided by either activated carbon or zeolite supports, tuneable acidity, and strong metal–ligand interactions have rendered them particularly effective in environments where gas composition fluctuates. However, recovery of captured NO_*x*_ as reusable nitric acid is less direct than in aqueous acid-absorption systems because NO_*x*_ is commonly retained as adsorbed species or solid nitrite and nitrate products, requiring sorbent regeneration or additional chemical processing if acid recovery is desired.

#### 6.2.2 Irreversible NO_*x*_ capture

Irreversible capture involves converting NO_*x*_ into stable solid products, typically nitrate or nitrite salts, rather than retaining NO_*x*_ through a readily reversible adsorption process. Alkaline solid sorbents are highly effective in this application by neutralizing HNO_3_ upon contact. Alkaline neutralization using calcium hydroxide, sodium bicarbonate, sodium carbonate, or magnesium oxide reacts with HNO_3_ fumes and/or NO_*x*_ to form solid nitrate salts.^19^ These reactivities may be enhanced by combining the sorbent with urea or carboxylic acid slurries.^99^ Typically delivered using dry sorbent injection (DSI), these sorbents are widely used in dry gas scrubbing systems, waste-treatment facilities, and fertilizer plants.

Metal oxides can also provide irreversible or strongly retained NO_*x*_ capture through formation of surface nitrate species. CuO, Fe_2_O_3_, WO_3_, and MnO_4_ are all examples that can react with NO_2_ to form nitrate species on the solid surface.^31^ These metals have also been prepared in TiO_2_–V_2_O_5_ blends, which are known for their robustness and thermal stability.^100^ The combined materials may also act as the active component in selective catalytic reduction (SCR) (Section 6.3) or as a low temperature NO_*x*_ removal catalyst.^101^ Depending on the formulation and operating conditions, such materials bridge irreversible capture and catalytic conversion, with their ultimate function determined by whether the surface nitrate species remain as captured products or participate in subsequent chemical reactions.

The primary advantage of irreversible solid capture is that NO_*x*_ can be removed without requiring continuous liquid circulation; however, irreversible capture consumes the reactive capacity of the sorbent, making sorbent replacement, maintenance, or disposal an important consideration. In addition, because NO_*x*_ is converted to solid nitrite and nitrate products, recovery of the nitrogen oxides as reusable nitric acid would be more difficult than in aqueous absorption systems or in using a reversible media.

### 6.3 Catalytic conversion of NO_*x*_

Catalytic conversion differs from both temporary storage and irreversible capture because the objective is to chemically transform NO_*x*_ into harmless byproducts, rather than retain it on the sorbent.^102^ Selective catalytic reduction of nitrogen oxides (NO_*x*_) is well known in emissions control systems transforming the NO_*x*_ to nitrogen (N_2_(g)) and water (H_2_O(g)) through the use of catalysts (*e.g.*, TiO_2_–WO_3_–V_2_O_5_, TiO_2_–MoO_3_–V_2_O_5_)^6,103,104^ or thermally^105^ with ammonia as the reductant. The SCR reactions can be broken down into different groups, depending on the mixture distributions of NO and NO_2_. First, the standard, dominant reaction when NO is the primary gas present is shown in eqn (13) where ammonia reacts with NO and oxygen to form N_2_ and H_2_O (assuming 1 : 1 stoichiometry for NH_3_ and NO).^104^ An alternative version of this first reaction under anoxic conditions is shown in eqn (14).^104^ Second, the SCR reaction shown in eqn (15) is prevalent when the concentrations of NO and NO_2_ are equimolar.^104^ If NO_2_ concentrations dominate, then eqn (16) is the dominant reaction.^104^ The optimal reaction temperatures for SCR is in the 190–260 °C range, although the reaction can proceed at higher or lower temperatures under the right conditions.

13 4NH_3_ + 4NO + O_2_ → 4N_2_ + 6H_2_O

14 4NH_3_ + 6NO → 5N_2_ + 6H_2_O

15 4NH_3_ + 2NO + 2NO_2_ → 4N_2_ + 6H_2_O

16 8NH_3_ + 6NO_2_ → 7N_2_ + 12H_2_O

One of the main issues with SCR is ammonia slip, where ammonia gas is inadvertently transported to and may foul downstream equipment. Ammonia slip can be mitigated with ammonia slip catalysts, referred to as ASC or ASX, examples of which include platinum metal group (PMG)-based supports (*e.g.*, Pt/TiO_2_, Pt/Al_2_O_3_, PMG-zeolites). In addition, a modification to SCR called selective non-catalytic reduction (SNCR) has been used but the efficiency is lower than SCR.^82^

Significant improvements in the catalyst efficiency have been obtained to mitigate environmental effects of NO_*x*_ with efficiencies reaching > 90%.^103,106^ Within UNF recycling, SCR may be used to mitigate the NO_*x*_ off gas from dissolvers or advanced head end processes, such as thermal fuel oxidation (FuelOx).^107^ Mitigation of NO_*x*_ in the preparation and dissolution of UNF minimizes the deleterious impacts from NO_*x*_ exposure onto other systems as described elsewhere and enables selective and efficient capture of other volatile radioisotopes.^57^

In late 2025, the waste treatment and immobilization plant (WTP), located on the Hanford site in southeastern Washington state, began processing low-activity radioactive waste (LAW) where SCR is used to treat NO_*x*_.^108^ The tank waste supernatant and salt cake is enriched in nitrates and nitrites. These components account for >90% of the waste volume but <10% of the activity. The tank sludge also contains nitrates and nitrites but much less than the supernatant and salt cake. The sludge accounts for <10% of the volume but >90% of the activity. Waste is mixed with glass-forming chemicals in a melter feed. When charged into the Joule-heated melter, thermal decomposition of nitrogen-bearing salts generates NO_*x*_ gases, which are directed into the primary and secondary off-gas systems. In the LAW vitrification facility, the primary off-gas system includes a submerged bed scrubber (SBS) and a wet electrostatic precipitator (WESP). These components serve various functions but also remove NO_*x*_ from the off-gas and transport it to the condensate vessels. In the secondary off-gas system, remaining NO_*x*_ is catalytically reduced through SCR to N_2_ by adding NH_3_(g) from the NH_3_ reagent facility. Efficient catalytic reduction is best achieved at high temperatures. A pre-heater system increases the temperature of inlet gas and heat generation of the catalytic reduction process itself is used to achieve the desired temperature. Excess ammonia is added to ensure robust operating conditions.

### 6.4 Membrane separation technologies

Membrane-based separations are being deployed in a growing range of industrial applications, which benefit from their intrinsic advantages in scalability, modularity, energy efficiency, and space efficiency. Membranes selectively separate gas streams continuously and without producing waste solutions or sorbents; only the membrane modules themselves must be disposed of at the end of their lifetime, which can be as long as several years.^109^ Despite these key benefits, the authors are not aware of previous work using membranes to separate NO_*x*_ at reprocessing facilities; even adjacent areas of research that could be used to predict membrane performance in this application remain mostly unexplored. However, two factors suggest that membranes for this purpose could be developed. First, a comparison of the physical properties of the off-gas components reveals differences that could be exploited by membranes to fractionate off-gas into streams that are more readily recycled or processed by capture technologies. Second, while membranes in reprocessing facilities would face significant material stability challenges, several recent advances in membrane science have produced membrane materials that tolerate other harsh environments; this trend may likewise lead to membranes that can survive reprocessing off-gas streams.^110–118^ In this section, we review the fundamental support for a hypothesis that membrane separations could enhance an overall reprocessing facility strategy for handling NO_*x*_ and radionuclides. We will then examine the specific obstacles to be overcome, and we will highlight and synthesize literature from disparate topic areas that provide some insight into performance gaps that remain and how they might be closed.

To understand how membranes could be deployed at nuclear reprocessing facilities, we must first determine what separations they might be able to achieve. Fig. 5 shows two properties of common off-gas components that are primary predictors of transport: boiling point, which tends to correlate with transport through rubbery polymer films, and kinetic diameter, which tends to be inversely correlated with transport through glassy polymer films and ceramics. Since the kinetic diameters of alkyl iodides,^119^ I_2_,^119^ and HI are larger than those of the dominant expected NO_*x*_ species (though not of N_2_O_4_),^120^ glassy polymers would tend to preferentially retain iodine species while permeating NO_*x*_, along with water and CO_2_; a ceramic membrane with sufficiently small pore size would behave similarly. The iodine-enriched retentate could then be better processed by NO_*x*_^−^ and water-sensitive iodine capture methods, and the NO_*x*_^−^ enriched permeate could be recycled upstream for reuse. If a rubbery polymer were used, the higher boiling point of I_2_ would suggest that its permeability would be higher than that of NO_*x*_, especially if NO is the dominant form; membrane permeability to alkyl iodides and HI would be similar to that for NO_2_. Depending on the dominant forms of iodine and NO_*x*_, an iodine-enriched permeate and NO_*x*_-enriched retentate could be generated. A cascade of membrane separations with different membrane materials used at different stages to produce more finely fractionated streams can also be easily envisioned. However, it must be emphasized that this analysis can give only a rough, preliminary idea of what streams could be obtained. Membrane perm-selectivity will also be influenced by the structure and chemistry of the membrane material and its interactions with off-gas molecules. Several additional factors must be considered to accurately predict the composition and flowrate of separated streams, including the dominant form of iodine and NO_*x*_ in the incoming off-gas stream (Section 3), the driving force for transport of each individual species (which is the difference in partial pressure across the membrane), and the ratio of membrane surface area to feed stream flowrate. Still, Fig. 5 can provide some ideas as to (1) which off-gas streams might best be separated by membranes of different material types and (2) how membranes could be combined with other capture and separation technologies. Given the dearth of existing data for membrane permeability to off-gas species under relevant conditions, we consider this to be an appropriate strategy for developing new research pathways.

**Fig. 5:**
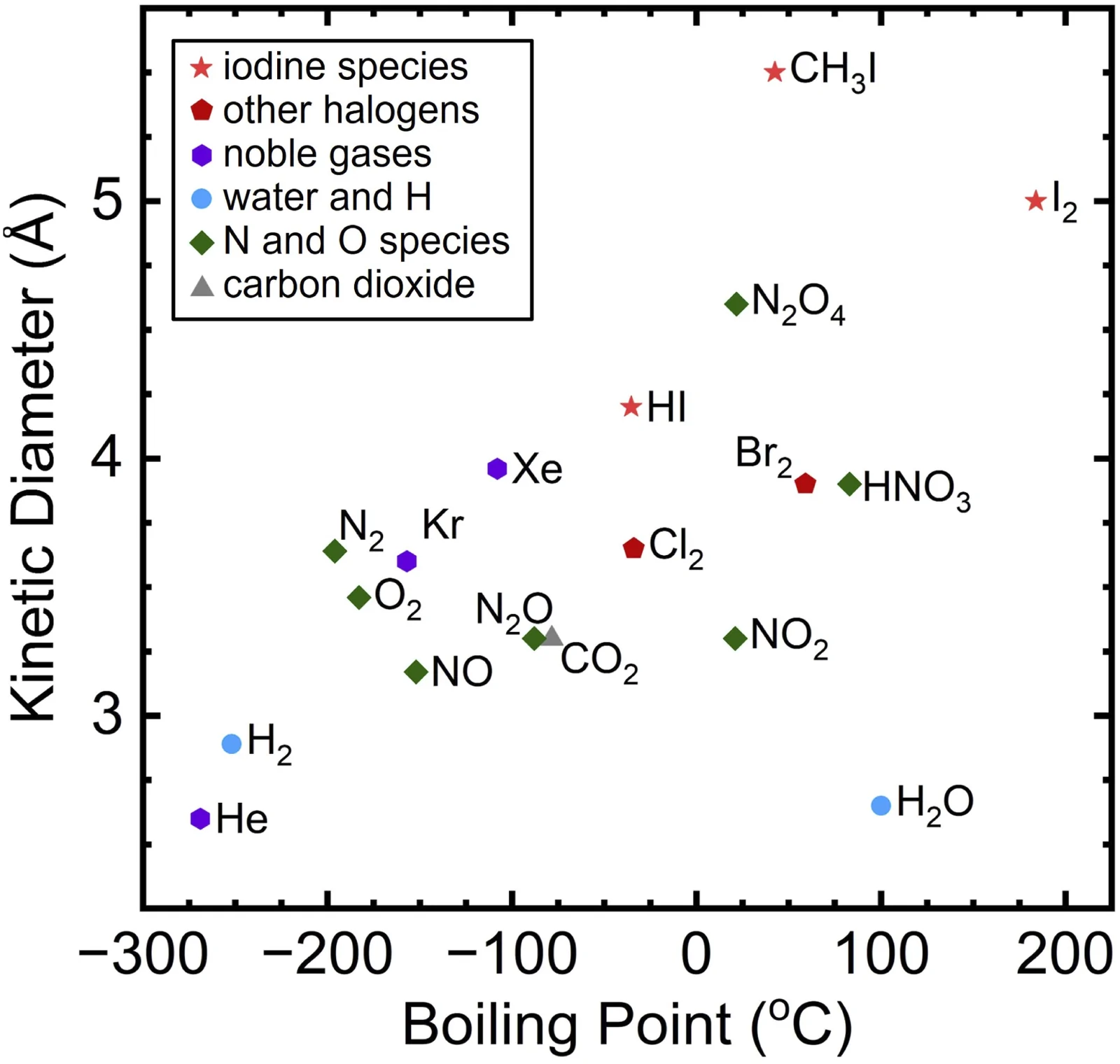
The kinetic diameter of various off-gas elements as a function of their boiling point. See Table S1 (SI) for data references.

Any membranes developed for this application will require resistance to degradation by NO_*x*_ and radiolysis. This challenge is significant, as polymers that are generally known for greater resilience to oxidation by NO_*x*_ (*e.g.*, fluorinated polymers) are distinct from those identified as being more robust against irradiation (*e.g.*, polyetheretherketone). Several literature reviews and reports have explored the robustness of polymers to both stressors, sometimes simultaneously, but often under conditions that are more mild than those relevant to reprocessing.^121–123^ Ultimately, experiments on membrane robustness that are conducted with representative off-gas compositions, radiation dose, temperature, and pressure during test-gas permeation will be required. Experimentally determining the expected lifetime of possible membrane materials is a critical step to understanding what other membrane performance metrics would be required to achieve economic viability. These other metrics include selectivity, permeability, cost of manufacturing for both membranes and modules, and cost of disposal. Data in all these areas is currently insufficient to set more specific targets; development largely remains at the conceptual stage.

A small body of data on membrane permeability to and stability in NO_*x*_ gas streams from non-nuclear applications can provide some limited insight into the gaps that remain to be overcome. The nearest commercial or pilot-scale application of membranes to separation of NO_*x*_ from facility off-gases is the separation of flue gases and natural gas. Although these focus on separation of CO_2_, H_2_S, H_2_O, or He,^124,125^ the gas streams tested can also contain significant concentrations of NO_*x*_. Reports typically do not include membrane perm-selectivity for NO_*x*_ species, but they do examine membrane stability in the presence of NO_*x*_. While most gas separation membranes in this application space require NO_*x*_ removal as a pretreatment step,^126^ stable operation of polymeric CO_2_-selective membranes has been demonstrated for membranes at bench and pilot scale with NO_*x*_ concentrations of 200 ppm and, for limited periods, up to 600 ppm.^127–130^ These NO_*x*_ concentrations are likely to be exceeded in reprocessing off-gas streams; substantial work remains to be done. A few studies have characterized membrane separation of NO, NO_2_, and N_2_O_4_ from other flue gas species. Scholes and coworkers^131^ measured the permeability of three glassy polymeric membranes to NO, along with CO_2_ and N_2_. The NO permeability was tested at 35 °C as 2500 ppm NO in N_2_ and was measured as 3.1, 3.7, and 168 Barrer in polysulfone, Matrimid 5218, and 6FDA-TMPDA membranes, respectively. In each of the three membranes, permeability to NO was lower than permeability to CO_2_ by a factor of 1.8, and higher than permeability to N_2_ by a factor of 3.6. These trends suggest that condensability of the off-gas species was a primary predictor of permeability in these specific membrane materials. A review by Pasichnyk and coworkers^116^ reports NO_*x*_ permeabilities for several additional types of membranes, some of which feature materials that are likely more radiolytically robust than polymers, *e.g.*, zeolites and ceramics. The drawback to these materials is that they may be more expensive and challenging to scale-up into manufacturable membrane modules.

Overall, while the properties of off-gas-species suggest the possibility for membrane separations to complement other NO_*x*_ and radionuclide technologies by separating off-gas into streams that are more easily recycled or processed, much research is needed to develop this potential opportunity. Direct measurements of membrane permeability to relevant species and membrane resilience under representative conditions must be obtained; these data can then be used to more precisely identify ideal pairings of membrane processes with other technologies and to determine performance requirements for economic viability.

### 6.5 NO_*x*_ detection methods

Continuous monitoring is necessary to ensure that NO_*x*_ controls are performing as intended. Processes for detecting and quantifying NO_*x*_ include chemiluminescence, non-dispersive infrared (NDIR) absorption, Fourier transform infrared (FTIR) spectroscopy, tunable diode laser absorption spectroscopy (TDLAS), electrochemical sensors, wet chemical methods, differential optical adsorption spectroscopy (DOAS), and mass spectrometry (MS), and are compared in Table 9. The choice of detection method depends on NO_*x*_ concentration, environment (including temperature, moisture, and other gas species present), importance of species differentiation, required response time, compatibility with regulatory requirements, commercial availability of instruments, and cost. Commercially available detection systems often combine multiple techniques to leverage the advantages of different methods.

**Table 9:** A summary of the specifications and advantages and disadvantages of different methods for NO_*x*_ gas detection

Method	Typical detection limit	Typical detection range	NO_*x*_ speciation	Typical response time	Advantages	Limitations	Typical cost
Chemiluminescence	∼0.1–5 ppb	0–1000 ppm	Yes, indirectly. Requires NO_2_ conversion to NO	∼5–300 s	Fast, continuous measurement; linear response; simple operation; widely used NO_*x*_ detection method	Requires conversion of NO_2_ to NO; potential for interference from other nitrogen compounds; potential interference from ozone	$$
Non-dispersive infrared (NDIR)	∼10–50 ppm	0–5000 ppm	Sometimes. Requires separate detection system for each species	∼10–20 s	Simple approach; continuous monitoring; moderate sensitivity; fast response	Potential interference from water vapor; worse signal to noise than narrowband spectroscopy techniques; not as commonly used for NO_*x*_ detection as other methods	$-$$
Fourier transform infrared spectroscopy (FTIR)	ppm^a^	ppm levels^a^	Yes, directly	∼10–60 s	Simultaneous multi-gas analysis	Expensive; detection limit and range are highly dependent on sample characteristics	$$-$$$
Tunable diode laser absorption spectroscopy (TDLAS)	<ppm^a^	ppm levels^a^	Yes. Requires separate configuration for each species	∼10 ms–3 s	Very fast response times; good species selectivity; functional in harsh conditions	Expensive; configuration is typically specific for a single species	$$-$$$
Wet chemical	ppm^a^	ppm levels^a^	Sometimes, detection typically requires NO oxidation to NO_2_	Depends on chosen sampling period (typically 10 min – 2 h) and analysis method	Samples may be stored for up to ∼48 hours until analysis	Slow response; low selectivity, especially in the presence of other nitrogen compounds	$
Electrochemical sensors	∼1–50 ppm	0–1000 ppm	Sometimes. Issues with NO and NO_2_ cross-selectivity are common	∼10–30 s	Low cost; simple implementation; can be suitable for harsh conditions like high humidity and elevated temperatures depending on the design	Limited lifetime; require regular calibration; NO and NO_2_ cross-selectivity; limited selectivity may preclude accurate measurement in complex gas streams	$
Differential optical absorption spectroscopy (DOAS)	<ppm^a^	ppm–%^a^	Yes, directly	∼1–2 min	Selective, simultaneous multi-gas analysis; often combined with FTIR to provide more analysis options	Species with overlapping absorption bands (such as SO_2_) will interfere with accurate measurement	$$
Mass spectrometry	<ppm^a^	ppm–%^a^	Sometimes. Requires specialized analysis or specific instrument selection to avoid NO and NO_2_ interference	<1–5 s	Fast response; selective, simultaneous multi-gas analysis	Expensive; species with a similar *m*/*z* will interfere with accurate measurement	$$-$$$

a Detection limits and ranges for these methods are highly dependent on instrumentation and sampling conditions.

The wide range of analytical techniques available for NO_*x*_ detection each have distinct advantages, limitations, and application niches. The NO_*x*_ detection capabilities of spectroscopy techniques (NDIR, FTIR, TDLAS, DOAS, MS) rely extensively on the chosen instrument configuration and measurement parameters, allowing for selections that are suitable for a specific application. Several spectroscopy techniques are suitable for NO_*x*_ differentiation in complex gas streams due to high spectral resolution (*e.g.*, FTIR). TDLAS systems can be configured for harsh measurement conditions (complex gas streams at high temperatures). Detection systems based on chemiluminescence and electrochemical sensors have been rigorously developed for simple implementation, are cost-effective, and are broadly commercially available. Several wet chemical methods have also been well-studied for NO_*x*_ gas quantification, although their slower response time makes it suited to supplementing other analysis techniques depending on the application. The performance of any gas sensor will depend on the measurement environment, as different detection methods are better suited to the certain types of harsh conditions (high temperature, high humidity, complex gas stream, presence of corrosive gas, *etc.*).^132^ Together, these methods cover detection limits from ppb to percent concentration levels, with different capabilities in terms of response time, robustness, species discrimination, and operational complexity, making method selection dependent on measurement goals, gas matrix, and practical constraints. Additional information for all these techniques is provided in Table 9.

## 7 Industrial case studies: what existing plants teach us about off-gas sequencing

In an aqueous UNF reprocessing plant, NO_*x*_ can be generated at many different parts of the process, each with unique off-gas challenges. The scrubbing of NO_*x*_ in reprocessing plants operating in the 21st century follow similar approaches. Here we examine the Thermal Oxide Reprocessing Plant (THORP) at Sellafield, UK; UP-2-800 and UP-3 at La Hague, France; and the Rokkasho Reprocessing Plant in Japan.^133,134^ These plants all scrub NO_*x*_ as the first major unit operation on the dissolver off-gas stream. A summary of these examples along with the Hanford WTP is presented herein (Table 10).

**Table 10:** A summary of international nuclear facilities implementing NO_*x*_ scrubbing. See text for more information

Plant	Location	Reported NO_*x*_ scrubbing technology used
THORP	Sellafield (United Kingdom)	Condensation, absorption and recombination in nitric acid and recycling as HNO_3_, homogenous oxidation of NO to NO_2_, and caustic scrubbing
UP2-800 and UP3	La Hague (Beaumont-Hague, France)	
Rokkasho Reprocessing Plant	Rokkasho (Aomori Prefecture, Japan)	
Hanford WTP	Richland, WA (USA)	SCR with NH_3_

### 7.1 Thermal oxide reprocessing plant (THORP)

At THORP, dissolver off-gas was treated in a series of operations to recover HNO_3_, abate NO_*x*_ and manage selected volatile radionuclides, including ^129^I and ^14^C (Fig. S1, SI).^134–136^ In the head end, NO_*x*_ was generated during UNF dissolution and later in the dissolver cycle to maintain Pu as Pu(iv) rather than Pu(vi) for downstream separation. Being a batch process, NO_*x*_ release was nonlinear and depended on where the dissolver was in the dissolution cycle. The largest release occurred when pieces of fuel were sheared and dropped into the dissolver, while later releases occurred during elevated-temperature dissolution and the NO_*x*_ conditioning step.

Abatement of the NO_*x*_ was achieved using a condenser, acid recombination column, plug flow reactor for NO to NO_2_ oxidation, and a caustic scrubber. The condenser and acid recombination column recovered HNO_3_ for use elsewhere in the process. Because NO is not particularly soluble and does not react with caustic liquors, the plug-flow reactor (*i.e.*, “noxidiser”) provided residence time for oxygen in the air supply to convert NO to NO_2_ and increase the decontamination factor of the caustic scrubber. Reported NO_*x*_ decontamination factors were 1.5 for the condenser, 3 for the acid recombination column, and 100 for the caustic scrubber, with an overall NO_*x*_ decontamination factor of 450.^135^

The product liquor from HNO_3_ scrubbing was treated with hot air to desorb iodine, and the gas from the reactor and heated scrubber was treated in a caustic scrubber that removed nitrogen dioxide, iodine, and carbon. The ^129^I was discharged to the sea, an option that may not be available depending on local regulations and geographic location. A portion of the caustic scrubber liquor was periodically purged, and carbon was precipitated by addition of barium nitrate as barium carbonate containing ^14^C, which was encapsulated in cement for disposal.

### 7.2 La Hague (France)

Similar to THORP, La Hague generates nitrogen oxides primarily during head-end shearing and HNO_3_ dissolution of spent oxide fuel. Unlike THORP's batch dissolver operation, the UP3 and UP2-800 plants use rotating-wheel dissolvers operated in a continuous mode, producing a more continuous dissolver off-gas stream,^4^ which is represented in Fig. 6.^23^ The NO/NO_2_ ratio generated during dissolution depends on HNO_3_ and nitrate concentrations, while residual iodine in the dissolver solution is further removed in an iodine desorber.^23^ Off gases from the dissolver and iodine desorber are treated by oxidation of nitrous vapours to HNO_3_, allowing the NO_*x*_ to be recovered as HNO_3_ for recycle. The treated gas is then combined with the vessel-vent gases from the dissolution units, and the combined stream is contacted with water in a packed absorption column. The stream then goes to an alkaline NaOH wet-scrubbing contactor, where volatile iodine is retained by conversion to non-volatile aqueous iodide and iodine oxyanions. Reports show that alkaline washing absorbs >96% of ^129^I from shearing/dissolution gases.^4,23^ Residual gaseous iodine is captured on silver-bearing adsorber beds, likely as AgI and/or AgIO_3_, while high-efficiency particulate air (HEPA) filtration removes radioactive aerosols before stack discharge.

**Fig. 6:**
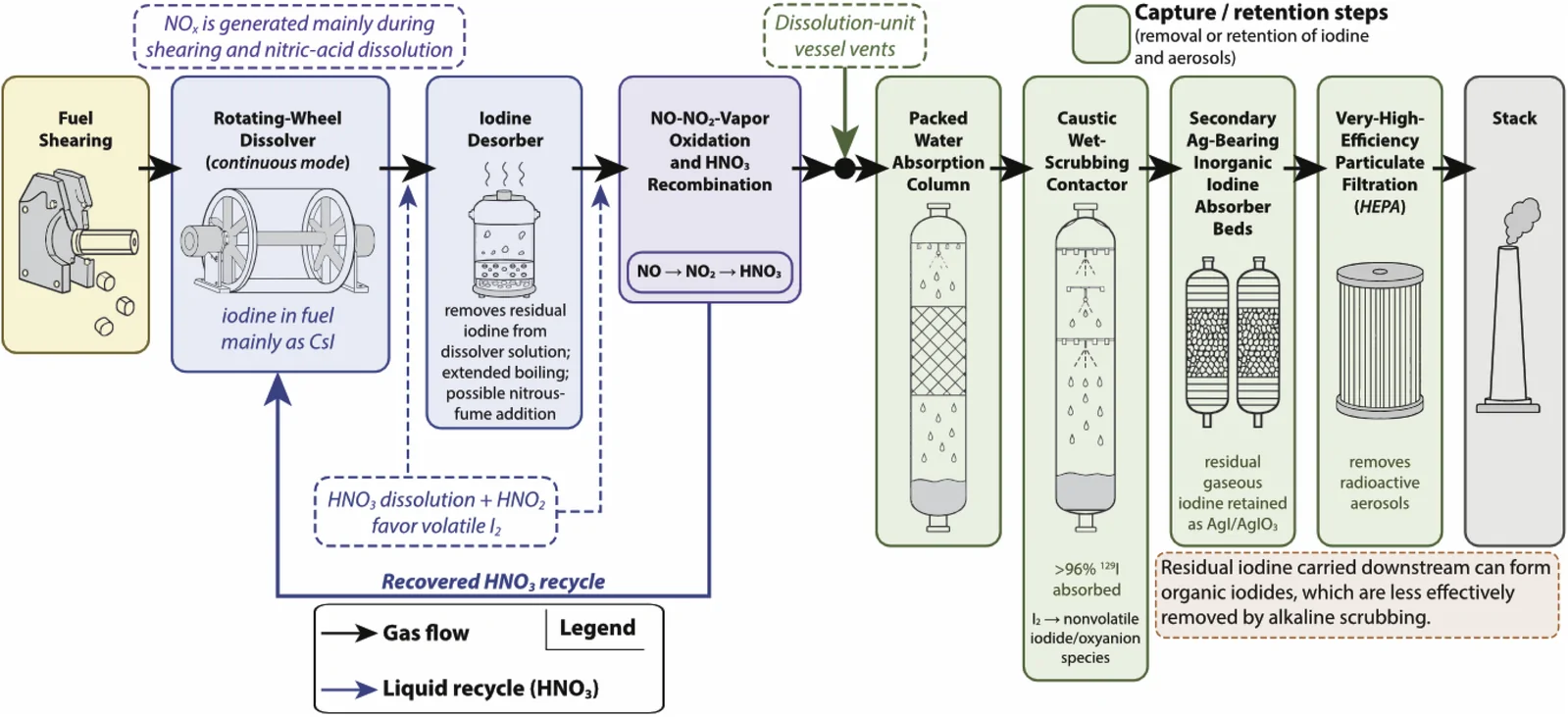
Flow diagram for La Hague off-gas treatment produced from descriptions found in references NEA/NSC/R(2022)4,^4^ DEN Monograph,^24^ and Meleshyn and Noseck.^23^ Portions of this image were generated with the assistance of OpenAI's ChatGPT and edited by the authors.

Iodine control at La Hague is strongly governed by speciation. Iodine is believed to enter dissolution primarily as CsI, but HNO_3_ dissolution and HNO_2_ chemistry favour oxidation to volatile I_2_, which is the preferred form for desorption into the dissolver off-gas.^23^ La Hague therefore uses an iodine desorber downstream of the rotating dissolver to minimize residual iodine in the dissolver solution. This is important because residual iodine can persist as iodate, organic iodides, or colloidal AgI/PdI_2_, and iodine carried into downstream solvent-extraction operations can form organic iodides that are poorly removed by alkaline wet scrubbing. In the head-end treatment system, volatile I_2_ is captured in NaOH solution primarily as non-volatile iodide and iodine oxyanion species, while residual gaseous iodine is retained on silver-bearing inorganic adsorbent media as AgI and/or AgIO_3_.

### 7.3 Rokkasho nuclear fuel reprocessing facility (Japan)

Rokkasho's off-gas treatment follows the La Hague head-end continuous dissolver concept, but with a different iodine-management strategy.^28,137^ The plant uses mechanical shearing followed by HNO_3_ dissolution in continuous rotary dissolvers. Because continuous operation leaves a residual iodine inventory in the dissolver solution, the solution is passed through iodine desorbers before clarification and solvent extraction. Off gases from the shearing machine, dissolver, and iodine desorbers are routed through several processes: NO_*x*_ scrubbing or absorption, demisting/heating, solid iodine filtration, and HEPA filtration before discharge through the main stack. Japan Nuclear Fuel Limited (JNFL) lists two-stage HEPA filtration and two-stage iodine-filter banks for the UNF shearing/dissolution off-gas system, with particle removal efficiencies >99.9% per HEPA stage and iodine removal >99.6%.^28^ Unlike La Hague, where caustic scrubbing captures iodine into a liquid effluent, at Rokkasho, iodine is captured on Ag-impregnated solid media, avoiding iodine discharge to sea.^134^ Vessel off-gas and vitrification off-gas are treated in separate systems using combinations of scrubbers, condensers or mist filters, HEPA filters, iodine filters, and, for vitrification, a ruthenium absorber.

Rokkasho's HNO_3_ recovery system is reported to draw on U.K./Sellafield-derived technology, using reduced-pressure evaporation and fractionation to recover reusable acid at lower operating temperatures, thereby reducing corrosion of process materials.^138^ At the time of this writing, Rokkasho remains in the final commissioning stage, with full operations scheduled for March 2027 at the time of this publication, thus conclusions should be treated as the most likely design-basis for final operation based on available literature on the plant.

## 8 Other radionuclide considerations

Other radionuclides, in the off-gas stream such as krypton, xenon, and tritium, also suffer from their own NO_*x*_ related complications. In particular, activated carbon has been established as a low-cost and effective material for krypton and xenon capture.^139,140^ Unfortunately, activated carbon undergoes an exothermic reaction with NO_*x*_ that poses a fire risk and limits its usefulness.^141^ As a result, metal–organic frameworks (MOFs) have emerged as a potentially promising alternative for krypton and xenon capture in UNF reprocessing.^142,143^ Nevertheless, the impact of NO_*x*_ and other off-gas constituents on these krypton/xenon adsorbing MOFs in simulated off-gas streams has yet to be investigated. Thus, to avoid the known and potential effects of NO_*x*_ on radionuclide capture, the removal of NO_*x*_ as a pretreatment step is desirable. Regardless of the material, the NO_*x*_ can cause absorbents to age prematurely and lose their original structures, particularly if humidity is present.^144^

## 9 Future outlook

The major challenges for the future are focused around minimizing NO_*x*_ release to the environment, minimizing NO_*x*_-induced degradation to unit operations (*e.g.*, radioiodine capture bed), employing effective and creative methods for selectively capturing NO_*x*_ from complex off-gas streams, and implementing efficient recycling options (*e.g.*, NO_2_) where possible. NO_*x*_ can be recycled and converted to reusable chemicals within the aqueous reprocessing voloxidation train (*e.g.*, NO_2_, HNO_3_) through the reactions described herein. Effective NO_*x*_ removal from the off-gas streams prior to the iodine solid sorbent beds could drastically improve the DFs and overall capacity of the iodine solid sorbent bed. The main limitation to this is that the NO_*x*_ capture method should not adversely affect speciation and convert target radionuclides to unwanted speciation (*e.g.*, I_2_ → IO_3_^−^) complicating downstream capture processes. This is a major challenge that requires additional study. More work is needed to assess effects of NO_*x*_ exposure on promising iodine solid sorbents, including Bi-based and Cu-based materials being tested to replace the more expensive (and hazardous) Ag-based sorbents that have been historically used in these unit operations.

Traditional material design methods, such as experimental iteration, while robust, lack the necessary throughput across a large design-space to efficiently down-select next-generation materials for NO_*x*_ capture. To expediate down-selection, several groups have begun to incorporate AI/ML methods to screen candidate material such as porous MOFs and zeolites.^145–148^ Although these studies are not specific to nuclear reprocessing off-gas, the methods developed could be applied directly to this use case in the near future.

## 10 Summary and conclusions

This paper summarizes the effective methods for capturing NO_*x*_ (namely NO and NO_2_) in nuclear facilities, it provides and overview of NO_*x*_ capture strategies in international flowsheets (*i.e.*, THORP in the UK, La Hague in France, and Rokkasho in Japan), the chemistry of NO_*x*_ interactions with different processes, as well as an overview of commercial NO_*x*_ detection and measurement technologies. International UNF reprocessing plants THORP, La Hague, and Rokkasho all illustrate abatement or recovery of NO_*x*_ immediately following fuel dissolution and upstream of volatile radionuclide capture; however, each plant differs in treatment. Designs across all three plants emphasize that dissolver off gas is the dominant operation for NO_*x*_ generation. However, other plant processes can also generate significant NO_*x*_ in their off gases that may require treatment for residual inorganic and organic iodides that are not efficiently removed by simple single step processes, such as caustic scrubbing.

Effective off-gas treatment for nuclear fuel reprocessing should be designed as an integrated control system with materials chemistry as a central design pillar. This includes effectively and efficiently managing all the volatile constituents in the off-gas stream with plant DFs meeting regulatory requirements, which can include NO_*x*_ as well as a range of radionuclides like radioiodine (*i.e.*, ^129^I, ^131^I), ^3^H (tritium), ^14^C, ^85^Kr, and ^106^Ru. In particular, NO_*x*_ constituents generated during nitric-acid dissolution can alter iodine speciation, react with getter materials, consume active binding sites, and accelerate sorbent aging under realistic process conditions. For iodine capture, this means that downstream bed performance cannot be separated from upstream NO_*x*_ management. Process design considerations such as removing or moderating NO_*x*_ before radioiodine solid sorbent beds may help maximize the DF performance of the solid sorbent bed, preserve iodine capacity, improve bed lifetime, reduce premature sorbent replacement, and help prevent the production of unwanted side reactions as NO_*x*_ interacts with the sorbents (*e.g.*, AgNO_3_ production, silver oxidation). The off-gas requirements of advanced processing options such as NO_2_ voloxidation should be included as part of the feasibility assessment for design and implementation. The range of NO_*x*_ capture technologies discussed herein include wet scrubbing methods (*i.e.*, caustic or acidic), solid sorbent methods (*e.g.*, activated carbon, zeolites, metal oxides), selective catalytic reduction, and several emerging membrane-based separations technologies. While NO_*x*_ removal technologies are mature, there remains opportunity for testing new ideas against some of the extreme conditions presented by advanced processing schemes.

In conclusion, the complexity of a reprocessing off gas feed induces limitations on controlling and capturing emissions. The presence of NO_*x*_ is not just a concentration problem, but rather, a holistic approach needs to be considered regarding the high complexity of the overall off-gas stream.

## Author contributions

Jonathan S. Evarts – conceptualization, writing – original draft, visualization, writing – review & editing. Brian J. Riley – conceptualization, writing – original draft, visualization, writing – review & editing. Stuart Arm – conceptualization, writing – original draft, writing – review & editing. Katherine R. Johnson – conceptualization, writing – original draft, visualization, writing – review & editing. Craig Milroy – writing – original Draft, writing – review & editing. Joanna McFarlane – conceptualization, writing – original draft, writing – review & editing. Krista Carlson – writing – original draft, visualization, writing – review & editing. Randy Ngelale – writing – original draft, writing – review & editing. Richard T. Mayes – writing – original draft, writing – review & editing. Laura Arias Chavez – writing – original draft, visualization, writing – review & editing. Alexander Wiechert – writing – original draft, writing – review & editing. Harmony S. Werth – writing – original draft, writing – review & editing. Joshua Turner – writing – original draft, writing – review & editing. Chase C. Cobble – writing – original draft. Jose Marcial – writing – original draft.

## Conflicts of interest

The authors declare no conflicts of interest.

## Supplementary Material

RA-OLF-D6RA08007C-s001

## Data Availability

No new data were created or analysed in this study. Data sharing is not applicable to this article. Supplementary information (SI) is available. See DOI: https://doi.org/10.1039/d6ra08007c.

## References

[cit1] Hao M., Liu X., Xie Y., Chen H., Gu H., Luo C., Xiao M., Zhang X., Li J., Yang X., Zhang J., Yang H., Fan F., Liang J., Wang X. (2026). Framework materials for nuclear waste treatment. Chem. Soc. Rev..

[cit2] Jubin R. , Spent Fuel Reprocessing, in Proceedings of the Introduction to Nuclear Chemistry and Fuel Cycle Separations Course, Consortium for Risk Evaluation with Stakeholder Participation (CRESP), 2008

[cit3] Jubin R. , Jordan J. A., Spencer B. B., Soelberg N., Welty A. K., Greenhalgh M., Strachan D., Thallapally P., Evaluation of an Integrated Off-gas System for a Used Nuclear Fuel Processing Facility, Proceedings of the GOBAL 2017, Seoul, Korea, 2017, vol. 200

[cit4] NEA , NEA/NSC/R(2022)4, Treatment of Volatile Fission Products, NEA Nuclear Science Committee, Paris, 2022.

[cit5] Soelberg N. R. , Jubin R. T., Strachan D. M., Ebert W. L., Greenhalgh M. R., Welty A. K., Greaney A. T., McFarlane J., Matyas J., Riley B. J. and Thallapally P. K., ANL/NSE-23/63; INL-RPT-23-74541; ORNL/SPR-2023/3084; PNNL-34867, Argonne National Laboratory, Lemont, IL, 2023.

[cit6] Chen Z., Liu Q., Liu H., Wang T. (2024). Recent Advances in SCR Systems of Heavy-Duty Diesel Vehicles—Low-Temperature NO_*x*_ Reduction Technology and Combination of SCR with Remote OBD. Atmosphere.

[cit7] Ghouma I., Jeguirim M., Sager U., Limousy L., Bennici S., Däuber E., Asbach C., Ligotski R., Schmidt F., Ouederni A. (2017). The Potential of Activated Carbon Made of Agro-Industrial Residues in NO_*x*_ Immissions Abatement. Energies.

[cit8] Helfritch D., Bortz S., Beittel R., Bergman P., Toole-O'Neil B. (1992). Combined SO_2_ and NO_*x*_ removal by means of dry sorbent injection. Environ. Prog..

[cit9] Lee Y.-W., Park J.-W., Yun J.-H., Lee J.-H., Choi D.-K. (2002). Studies on the Surface Chemistry Based on Competitive Adsorption of NO_*x*_-SO_2_ onto a KOH Impregnated Activated Carbon in Excess O_2_. Environ. Sci. Technol..

[cit10] Chen Z. , Liu Q., Liu H. and Wang T., Recent Advances in SCR Systems of Heavy-Duty Diesel Vehicles—Low-Temperature NO_*x*_ Reduction Technology and Combination of SCR with, Remote OBD, 2024

[cit11] Ghouma I. , Jeguirim M., Sager U., Limousy L., Bennici S., Däuber E., Asbach C., Ligotski R., Schmidt F. and Ouederni A., The Potential of Activated Carbon Made of Agro-Industrial Residues in NO_x_ Immissions Abatement, 2017.

[cit12] Patsias A. A., Nimmo W., Gibbs B. M., Williams P. T. (2005). Calcium-based sorbents for simultaneous NOx/SOx reduction in a down-fired furnace. Fuel.

[cit13] Chen J.-C., Liu Z.-S., Huang J.-S. (2007). Emission characteristics of coal combustion in different O_2_/N_2_, O_2_/CO_2_ and O_2_/RFG atmosphere. J. Hazard. Mater..

[cit14] Guimerà X., Mora M., Dorado A. D., Bonsfills A., Gabriel D., Gamisans X. (2021). Optimization of SO_2_ and NO_x_ sequential wet absorption in a two-stage bioscrubber for elemental sulphur valorisation. Environ. Sci. Pollut. Res..

[cit15] Birdwell J. , Iodine and NOx behavior in the dissolver off-gas and IODOX [Iodine Oxidation] systems in the Oak Ridge National Laboratory Integrated Equipment Test facility, Proceedings of the 21st DOE/NRC Nuclear Air Cleaning Conference, San Diego, CA, 1990, pp. CONF–900809–900801

[cit16] Johnson K. R., Greaney A. T., Peruski K. M., Cobble C., Vick M. C., Glasgow D., McFarlane J. (2026). NO_2_-mediated voloxidation for iodine separation from cesium iodide surrogates. Sep. Sci. Technol..

[cit17] Bruffey S. H. , Jubin R. T., Anderson K. K., Walker J. and Franklin J., Aging and Iodine Loading of Silver-Functionalized Aerogels, in Proceedings of the Global 2013, DE–AC05–00OR22725, 2013

[cit18] Bruffey S. H. , Patton K. K. and Jubin R. T., ORNL/LTR-2015/258; AF5805010; NEAF327, Oak Ridge National Laboratory, Oak Ridge, TN, 2015

[cit19] Bruffey S. H. , Anderson K. K., Walker Jr J. F. and Jubin R. T., FCRD-SWF-2013-000258, Oak Ridge National Laboratory, Oak Ridge, TN, 2013.

[cit20] Patton K. K. , Bruffey S. H., Jubin R. T. and Walker J. F., ORNL/LTR-2014/42, Oak Ridge National Laboratory, Oak Ridge, TN, 2014.

[cit21] Patton K. K. , Bruffey S. H., Walker J. F. and Jubin R. T., ORNL/LTR-2014/271, Oak Ridge National Laboratory, Oak Ridge, TN, 2014.

[cit22] Carlson K., Riley B. J., Turner J., Cantu D. C., Chidambaram D., Lonergan C., Rimer J. D. (2025). Dual-Bed Radioiodine Capture from Complex Gas Streams with Zeolites: Regeneration and Reuse of Primary Sorbent Beds for Sustainable Waste Management. ACS Sustain. Chem. Eng..

[cit23] Meleshyn A. and Noseck U., GRS-294. Gesellschaft für Anlagen Und Reaktorsicherheit (GRS) mbH, Cologne, Germany, 2013.

[cit24] Abonneau E. , Baron P., Berthon C., Berthon L., Beziat A., Bisel I., Bonin L., Bosse E., Boullis B., Broudic J., Charbonnel M. C., Chauvin N., Den Auwer C., Dinh B., Duhamet J., Escleine J. M., Grandjean S., Guilbaud P., Guillaneux D., Guillaumont D., Hill C., Lacquement J., Masson M., Miguirditchian M., Moisy P., Pelletier M., Ravenet A., Rostaing C., Royet V., Ruas A., Simoni E., Sorel C., Vaudano A., Venault L., Warin D., Zaetta A., Pradel P., Bonin B., Bouquin B., Dozol M., Lecomte M., Forestier A., Beauvy M., Berthoud G., Defranceschi M., Ducros G., Guerin Y., Latge C., Limoge Y., Madic C., Santarini G., Seiler J. M., Sollogoob P., Vernaz E., Bazile F., Parisot J. P., Finot P. and Roberts J. F., Commissariat a l'energie atomique-(CEA), France, 2008.

[cit25] Baskaran K., Elliott C., Ali M., Moon J., Beland J., Cohrs D., Chong S., Riley B. J., Chidambaram D., Carlson K. (2023). Effects of NO_2_ aging on bismuth nanoparticles and bismuth-loaded silica xerogels for iodine capture. J. Hazard. Mater..

[cit26] Bion N. , Can F., Courtois X. and Duprez D., Transition metal oxides for combustion and depollution processes, in Metal Oxides in Heterogeneous Catalysis, ed. J. C. Védrine. Elsevier, 2018, pp. 287–353.

[cit27] Sakuragi T., Yoshida S., Kato O. (2023). Characteristics of Iodine-Bearing Silver-Impregnated Alumina Sorbents and Their
Direct Solidification *via* Hot Isostatic Pressing. Front. Chem..

[cit28] Japan Nuclear Fuel Limited , Rokkasho Reprocessing Plant, Aomori, Japan, 2014.

[cit29] Agency UEP , Review of the primary national ambient air quality standards for oxides of nitrogen, Federal Register, 2018, vol. 83, pp. 26752–26785

[cit30] New Source Performance Standards Review for Nitric Acid Plants, 77 Fed. Reg. 48433 (Aug. 14, 2012) (Codified at 40 C.F.R. pt. 60)

[cit31] Alves L., Holz L. I. V., Fernandes C., Ribeirinha P., Mendes D., Fagg D. P., Mendes A. (2022). A comprehensive review of NO_*x*_ and N_2_O mitigation from industrial streams. Renewable Sustainable Energy Rev..

[cit32] Bhowmick S., Badiwal A., Shenoy K. T. (2023). Removal of NO_*x*_ using ozone injection and subsequent absorption in water. Chem. Eng. J. Adv..

[cit33] Chmielewski A. G. (2007). Industrial applications of electron beam flue gas treatment—From laboratory to the practice. Rad. Phys. Chem..

[cit34] Nash K. L. and Nilsson M., Introduction to the reprocessing and recycling of spent nuclear fuels, in Reprocessing and Recycling of Spent Nuclear Fuel, ed. R. Taylor, Woodhead Publishing, Oxford, 2015, pp. 3–25.

[cit35] Hermann B. , Dissolution of Unirradiated UO_2_-Pellets in Nitric Acid. Technical Document Ref. 3673, Karlsruher Institut für Technologie Germany, 1984.

[cit36] Marc P., Magnaldo A., Vaudano A., Delahaye T., Schaer É. (2017). Dissolution of uranium dioxide in nitric acid media: what do we know?. EPJ Nuclear Sci. Technol..

[cit37] Abel E., Schmid H. (1928). The kinetics of nitrous acids. I, Introduction and overview & II, orienting experiments. Z. Phys. Chem..

[cit38] Abel E., Schmid H. (1928). Kinetics of decomposition of nitrous acid. Z. Phys. Chem..

[cit39] Abel E., Schmid H. (1929). Kinetics of nitrous acid IV, equilibrium of nitrous acid-nitric oxide reaction in conjunction with its kinetics. Z. Phys. Chem..

[cit40] Counce R. M., Perona J. J. (1983). Scrubbing of gaseous nitrogen oxides in packed towers. AlChE J.

[cit41] Makhotkin A., Shamsutdinov A. (1976). A Study of the Kinetics of the Absorption of NO_2_ and the Effect of Nitrous Acid. Khim. Khim. Tekhnol..

[cit42] Komiyama H., Inoue H. (1978). Reaction and Transport of Nitrogen Oxides in Nitrous Acid Solutions. J. Chem. Eng. Jpn..

[cit43] Kaiser E., Wu C. (1977). A kinetic study of the gas phase formation and decomposition reactions of nitrous acid. J. Phys. Chem..

[cit44] England C., Corcoran W. H. (1975). The rate and mechanism of the air oxidation of parts-per-million concentrations of nitric oxide in the presence of water vapor. Ind. Eng. Chem. Res..

[cit45] Wayne L. G., Yost D. M. (1951). Kinetics of the Rapid Gas Phase Reaction between NO, NO_2_, and H_2_O. J. Chem. Phys..

[cit46] Lefers J. B., de Boks F. C., van den Bleek C. M., van den Berg P. J. (1980). The oxidation and absorption of nitrogen oxides in nitric acid in relation to the tail gas problem of nitric acid plants. Chem. Eng. Sci..

[cit47] Beck C. L., Cervantes J., Chiswell S., Greaney A. T., Johnson K. R., Levitskaia T. G., Martin L. R., McDaniel G., Noble S., Rakos J. M., Riley B. J., Ritzmann A., Tingey J. M. (2024). Review of iodine behavior from nuclear fuel dissolution to environmental release. RSC Adv..

[cit48] Vienna J. D. , Collins E. D., Crum J. V., Ebert W. L., Frank S. M., Garn T. G., Gombert D., Jones R., Jubin R. T., Maio V. C., Marra J. C., Matyas J., Nenoff T. M., Sevigny G. J., Soelberg N. R., Strachan D. M., Thallapally P. K. and Westsik J. H., Closed Fuel Cycle Waste Treatment Strategy, PNNL-24114; TRN: US2301522, Pacific Northwest National Lab. (PNNL), Richland, WA, United States, 2015.

[cit49] Johnson J. A., Rawn C. J., Spencer B. B., Meisner R. A., Del Cul G. D. (2017). Oxidation kinetics for conversion of U_3_O_8_ to ε-UO_3_ with NO_2_. J. Nucl. Mater..

[cit50] Peruski K. M., Spano T. L., Vick M. C., Cobble C., Greaney A. T., McFarlane J. (2024). Elucidating the Composition and Structure of Uranium Oxide Powders Produced *via* NO_2_ Voloxidation. ACS Omega.

[cit51] Goode J. , Voloxidation: removal of volatile fission products from spent LMFBR fuels, ORNL-TM-3723, Oak Ridge National Laboratory, Oak Ridge, TN, 1973.

[cit52] Uchiyama G. , Yamazaki K., Sugikawa S., Maeda M., Tsujino T., Kitamura M. and Torikai S., Study on Voloxidation Process for Tritium Control in Reprocessing, JAERI-M-91-199, JP9202180, Japan Atomic Energy Research Institute, Tokyo, Japan, 1992

[cit53] Jubin R. T. , Strachan D. M. and Soelberg N. R., Iodine Pathways and Off-Gas Stream Characteristics for Aqueous Reprocessing Plants–A Literature Survey and Assessment, INL/EXT-13-30119, Idaho National Laboratory, Idaho Falls, ID, 2013

[cit54] Riley B. J., Turner J. R., McFarlane J., Chong S., Carlson K., Matyáš J. (2024). Iodine solid sorbent design: a literature review of the critical criteria for consideration. Mater. Adv..

[cit55] Azambre B., Chebbi M. (2017). Evaluation of Silver Zeolites Sorbents Toward Their Ability to Promote Stable CH_3_I Storage as AgI Precipitates. ACS Appl. Mater. Interfaces.

[cit56] Shen Z., Wiechert A. I., Choi S., Ladshaw A. P., Tavlarides L. L., Tsouris C., Yiacoumi S. (2025). Influence of elevated temperature and oxygen on the capture of radioactive iodine by silver functionalized silica aerogel. Microporous Mesoporous Mater..

[cit57] Greaney A. T., Ngelale R. O., Bruffey S. H., Martin L. R. (2023). Abatement of radioiodine in aqueous reprocessing off-gas. Front. Chem..

[cit58] Matyáš J., Ilton E. S., Kovařík L. (2018). Silver-functionalized silica aerogel: towards an understanding of aging on iodine sorption performance. RSC Adv..

[cit59] Shen Z., Wiechert A. I., Choi S., Ladshaw A. P., Tavlarides L. L., Tsouris C., Yiacoumi S. (2022). Silver-functionalized silica aerogel for iodine capture: Adsorbent aging by NO_2_ in spent nuclear fuel reprocessing off-gas. Microporous Mesoporous Mater..

[cit60] Wiechert A. I., Ladshaw A. P., Moon J., Abney C. W., Nan Y., Choi S., Liu J., Tavlarides L. L., Tsouris C., Yiacoumi S. (2020). Capture of Iodine from Nuclear-Fuel-Reprocessing Off-Gas: Influence of Aging on a Reduced Silver Mordenite Adsorbent after Exposure to NO/NO_2_. ACS Appl. Mater. Interfaces.

[cit61] Jubin R. , Ramey D., Spencer B., Anderson K. and Robinson S., Proceedings of the Waste Management 2012 Conference, Tempe, AZ, 2012

[cit62] Choi S., Nan Y., Tavlarides L. L. (2021). Kinetics of aging process on reduced Ag exchanged mordenite in dry air and humid air. AIChE J..

[cit63] Choi S., Nan Y., Tavlarides L. L. (2021). Aging kinetics on silver-functionalized silica aerogel in off-gas streams including dry air, humid air, NO and NO_2_. Chem. Eng. J. Adv..

[cit64] Patton K. K. , Bruffey S. H., Walker J. F. and Jubin R. T., ORNL/LTR-2014/271; M3FT-14OR03120210; FCRD-SWF-2014-000274, Oak Ridge National Lab. (ORNL), Oak Ridge, TN (United States), United States, 2014

[cit65] Patton K. K. , Bruffey S. H., Jubin R. T. and Walker J. F., ORNL/LTR-2014/425; R&D Project: AF5805010; FCRD-SWF-2014-000277, Oak Ridge National Lab. (ORNL), Oak Ridge, TN (United States), United States, 2014

[cit66] Bruffey S. , Patton K., Walker J. and Jubin R., Complete NO and NO_2_ Aging Study for AgZ, ORNL/SPR-2015/128, Oak Ridge National Lab. (ORNL), Oak Ridge, TN (United States), 2015

[cit67] Jordan J. A. and Bruffey S. H., FCRD-MRWFD-2016-000358, Oak Ridge National Laboratory (ORNL), Oak Ridge, TN (United States), 2016.

[cit68] Matyas J., Canfield N., Sulaiman S., Zumhoff M. (2016). Silica-based waste form for immobilization of iodine from reprocessing plant off-gas streams. J. Nucl. Mater..

[cit69] Chen G., Wu L., Wang Z., Liu R., Tan C., Tan Y., Liu Y., He M., Zhang H., Huang Y., Zhu J., Duan T., Zhu L. (2025). Aluminum-based MOF for efficient iodine and methyl iodide capture from NO_2_-containing gas streams. Chem. Eng. J..

[cit70] Eisawi K., Loni E., Chong S., Liezers M., Tang M., Brinkman K. S., Riley B. J., Naguib M. (2025). Nanohybrid of Silver-MXene: A Promising Sorbent for Iodine Gas Capture from Nuclear Waste. Adv. Mater. Interfaces.

[cit71] Lere-Adams A. J., Dixon Wilkins M. C., Bollinger D., Stariha S., Farzana R., Dayal P., Gregg D. J., Chong S., Riley B. J., Heiden Z. M., McCloy J. S. (2024). Glass-bonded ceramic waste forms for immobilization of radioiodine from caustic scrubber wastes. J. Nucl. Mater..

[cit72] Spencer B. , Bruffey S., Walker Jr J., Anderson K. K. and Jubin R., FCRD-SWF-2013-000329, Oak Ridge National Laboratory, Oak Ridge, TN, 2013

[cit73] Johnson K. , Cobble C., Dye C., Lawson K., Vick M., Riley B., Chong S. and Worth H., Oak Ridge National Laboratory (ORNL), Oak Ridge, TN (United States), 2025.

[cit74] Bruffey S. H. , Spencer B. B., Strachan D. M., Jubin R. T., Soelberg N. R. and Riley B. J., INL/EXT-15-35609, Idaho National Laboratory, Idaho Falls, ID, 2015.

[cit75] Johnson K. R., Greaney A. T., Peruski K. M., Cobble C., Vick M., McFarlane J. (2024). Influences of Iodine Separation and Capture in the Context of NO_2_-Based Voloxidation. Trans. Am. Nucl. Soc..

[cit76] Sakurai T., Takahashi A., Ishikawa N., Komaki Y., Ohnuki M., Adachi T. (1992). The iodine species and their behavior in the dissolution of spent-fuel specimens. Nucl. Technol..

[cit77] Burch W. D. , Bigelow J. E. and King L. J., Transuranium Processing Plant Semiannual Report of Production, Status, and Plans for Period Ending June 30, 1969, ORNL-4540, Oak Ridge National Laboratory, Oak Ridge, TN, 1970.

[cit78] Asmussen R. M., Westesen A., Cordova E. A., Fujii Yamagata A. L., Schonewill P. P., Moore A. C., Bourchy A., Saslow S. A., Smith G. L., Riley B. J., Skeen R. S. (2023). Iodine Removal from Carbonate-Containing Alkaline Liquids Using Strong Base Resins, Hybrid Resins, and Silver Precipitation. Ind. Eng. Chem. Res..

[cit79] Haefner D. R. and Tranter T. J., Methods of gas phase capture of iodine from fuel reprocessing off-gas: a literature survey, INL/EXT-07-12299, Idaho National Laboratory, Idaho Falls, ID, 2007.

[cit80] Holladay D. , Literature survey: methods for the removal of iodine species from off-gases and liquid waste streams of nuclear power and nuclear fuel reprocessing plants, with emphasis on solid sorbents, ORNL/TM-6350, Oak Ridge National Laboratory, Oak Ridge, TN, 1979.

[cit81] Patwardhan J. A., Pradhan M. P., Joshi J. B. (2002). Simulation of NO_*x*_ gas absorption under adiabatic condition and comparison with plant data. Chem. Eng. Sci..

[cit82] Ghriss O., Ben Amor H., Jeday M.-R., Thomas D. (2019). Nitrogen oxides absorption into aqueous nitric acid solutions containing hydrogen peroxide tested using a cables-bundle contactor. Atmos. Pollut. Res..

[cit83] Chu H., Chien T.-W., Twu B.-W. (2001). The absorption kinetics of NO in NaClO_2_/NaOH solutions. J. Hazard. Mater..

[cit84] Brogren C., Karlsson H. T., Bjerle I. (1997). Absorption of NO in an alkaline solution of KMnO_4_. Chem. Eng. Technol..

[cit85] Omar K. , Evaluation of BOC'S LoTOx Process for the Oxidation of Elemental Mercury in Flue Gas from a Coal-Fired Boiler, WRI-08-RO10, University of Wyoming Research Corporation, Laramie, Wyoming, 2008.

[cit86] Kartohardjono S., Merry C., Rizky M. S., Pratita C. C. (2019). Nitrogen oxide reduction through absorbent solutions containing nitric acid and hydrogen peroxide in hollow fiber membrane modules. Heliyon.

[cit87] Sada E., Kumazawa H., Kudo I., Kondo T. (1978). Absorption of NO in aqueous mixed solutions of NaClO_2_ and NaOH. Chem. Eng. Sci..

[cit88] Sada E., Kumazawa H., Yamanaka Y., Kudo I., Kondo T. (1978). Kinetics of absorption of sulfur dioxide and nitric oxide in aqueous mixed solutions of sodium chlorite and sodium hydroxide. J. Chem. Eng. Jpn..

[cit89] Brogren C., Karlsson H. T., Bjerle I. (1998). Absorption of NO in an Aqueous Solution of NaClO_2_. Chem. Eng. Technol..

[cit90] Hsu H. W., Lee C. J., Chou K. S. (1998). Absorption of NO by NaClO_2_ solution: Performance characteristics. Chem. Eng. Comm..

[cit91] Pourmohammadbagher A., Jamshidi E., Ale-Ebrahim H., Dabir S. (2011). Study on Simultaneous Removal of NO_*x*_ and SO_2_ with NaClO_2_ in a Novel Swirl Wet System. Ind. Eng. Chem. Res..

[cit92] Jin D.-S., Deshwal B.-R., Park Y.-S., Lee H.-K. (2006). Simultaneous removal of SO_2_ and NO by wet scrubbing using aqueous chlorine dioxide solution. J. Hazard. Mater..

[cit93] Sparks T. and Chase G., Section 3 - Air and Gas Filtration, in Filters and Filtration Handbook, ed. T. Sparks and G. Chase, Butterworth-Heinemann, Oxford, 6th edn, 2016, pp. 117–198.

[cit94] Tao H., Liu Y. (2023). Dynamic Adsorption/Desorption of NO_*x*_ on MFI Zeolites: Effects of Relative Humidity and Si/Al Ratio. Nanomaterials.

[cit95] Chen G., Gao J., Gao J., Du Q., Fu X., Yin Y., Qin Y. (2010). Simultaneous Removal of SO_2_ and NO_*x*_ by Calcium Hydroxide at Low Temperature: Effect of SO_2_ Absorption on NO_2_ Removal. Ind. Eng. Chem. Res..

[cit96] Sun Z., Liao Y., Zhang Y., Sun S., Kan Q., Wu Z., Yu L., Dong Z., Wang Z., He R., Wang L., Meng Q., Wang H., Wang Q., Mao L., Pan D., Wang S., Zhang Z., Zhu W., Liu S., Wakeel M., Hu B., Duan T., Tai X., Wang X. (2025). Sustainable carbon materials in environmental and energy applications. Sustainable Carbon Mater..

[cit97] Liu Y., Wu X., Yang X., Tao H., Li J., Zhang C., Yang R. T., Li Z. (2023). Enhancement of NO_*x*_ adsorption performance on zeolite *via* a facile modification strategy. J. Hazard. Mater..

[cit98] Iwasaki M., Shinjoh H. (2010). A comparative study of “standard”, “fast” and “NO_2_” SCR reactions over Fe/zeolite catalyst. Appl. Catal., A.

[cit99] Tyupina E. A., Pryadko A. V. (2024). Use of silver-containing sorbents in anionic species of radioactive iodine management in nuclear industry and the methods of obtaining them. J. Radioanal. Nucl. Chem..

[cit100] Arfaoui J., Ghorbel A., Petitto C., Delahay G. (2018). Novel V_2_O_5_-CeO_2_-TiO_2_-SO_4_^2−^ nanostructured aerogel catalyst for the low temperature selective catalytic reduction of NO by NH_3_ in excess O_2_. Appl. Catal., B.

[cit101] Yu J., Li C., Guo F., Gao S., Zhang Z. G., Matsuoka K., Xu G. (2018). The pilot demonstration of a honeycomb catalyst for the DeNOx of low-temperature flue gas from an industrial coking plant. Fuel.

[cit102] Wejkowski R., Kalisz S., Tymoszuk M., Ciukaj S., Maj I. (2021). Full-Scale Investigation of Dry Sorbent Injection for NO_*x*_ Emission Control and Mercury Retention. Energies.

[cit103] Han L., Cai S., Gao M., Hasegawa J.-y., Wang P., Zhang J., Shi L., Zhang D. (2019). Selective Catalytic Reduction of NO_*x*_ with NH_3_ by Using Novel Catalysts: State of the Art and Future Prospects. Chem. Rev..

[cit104] Koebel M., Elsener M., Kleemann M. (2000). Urea-SCR: a promising technique to reduce NO_*x*_ emissions from automotive diesel engines. Catal. Today.

[cit105] Lewis L. and Kruger A., 24590-CD-POC-MBT0-00007-02-00001_00E, Hanford Waste Treatment and Immobilization Plant, Richland, WA, 2015.

[cit106] Ismagilov Z. R., Kerzhentsev M. A., Shkrabina R. A., Tsikoza L. T., Lunyushkin B. I., Ostrovski Y. V., Kostin A. L., Abroskin I. Y., Malyi Y. N., Matyukha V. A., Adamson M. G., Heywood A. C., Zundelevich Y., Ismagilov I. Z., Babko-Malyi S., Battleson D. M. (2000). A role of catalysis for the destruction of waste from the nuclear industry. Catal. Today.

[cit107] Rodene D. D. , Marin Bernardez E. J., Gordon M. N., Lavenue M., Ramsey A. A., Garner S. A., Gilbraith W. E., Jiang J., Craps M. G., Adams W. T., Fushimi R., Chau H., Lauterbach J., Stofik S., Tong J., Zhou T., Aidhy D. S., Mueller M., Chen F., Qin B., Aravelli A. and Hobbs D., Electrochemical Denitration and Caustic Generation (EDCGe) System: Year One Project Report, SRNL-STI-2025-00233, Savannah River National Laboratory, Aiken, SC, 2025.

[cit108] Marcial J., Riley B. J., Kruger A. A., Lonergan C. E., Vienna J. D. (2024). Hanford low-activity waste vitrification: A review. J. Hazard. Mater..

[cit109] Bernardo G., Mendes A. (2026). Advances in O_2_/N_2_ separation membranes: A 25-year performance perspective. Sep. Purif. Technol..

[cit110] Yu S., Lin R., Xie Z., Chai M., Chen R., Li S., Shi H., Zhang K., Shi Z., Hou J. (2024). Recent membrane separation technology for noble gas recovery. J. Mater. Chem. A.

[cit111] Puspasari T., Wang Y., Ghanem B., Hu X., Elahi F., Wehbe N., Pinnau I. (2025). Development of High Performance Pyrolized Polyimide-based Carbon Molecular Sieves for Enhanced Selectivity of Propylene/Propane Gas Separation. Angew. Chem., Int. Ed..

[cit112] Liu Z., Cao Y., Koros W. J. (2025). Synergistic Tuning of Microstructure and Morphology in Carbon Molecular Sieve Hollow Fibers for Propylene/Propane Separation. Angew. Chem., Int. Ed..

[cit113] Marchetti P., Jimenez Solomon M. F., Szekely G., Livingston A. G. (2014). Molecular Separation with Organic Solvent Nanofiltration: A Critical Review. Chem. Rev..

[cit114] Aristizábal S. L., Lively R. P., Nunes S. P. (2023). Solvent and thermally stable polymeric membranes for liquid molecular separations: Recent advances, challenges, and perspectives. J. Membr. Sci..

[cit115] Lee J., Shin Y., Boo C., Hong S. (2023). Performance, limitation, and opportunities of acid-resistant nanofiltration membranes for industrial wastewater treatment. J. Membr. Sci..

[cit116] Pasichnyk M., Stanovsky P., Polezhaev P., Zach B., Šyc M., Bobák M., Jansen J. C., Přibyl M., Bara J. E., Friess K., Havlica J., Gin D. L., Noble R. D., Izák P. (2023). Membrane technology for challenging separations: Removal of CO_2_, SO_2_ and NO_*x*_ from flue and waste gases. Sep. Purif. Technol..

[cit117] Burnea L. C., Zaharescu T., Dumitru A., Plesa I., Ciuprina F. (2014). Radiation stability of polypropylene/lead zirconate composites. Rad. Phys. Chem..

[cit118] Wang W., Zhou M., Yang H., Shao Z. (2024). Radiation resistance of graphene in tritiated water. Fusion Eng. Des..

[cit119] Scheele R. D. , Burger L. L. and Matsuzaki C. L., Methyl Iodide Sorption by Reduced Silver Mordenite,, PNL-4489, Pacific Northwest National Laboratory, Richland, WA, 1983.

[cit120] Liu Y., Zuo J., Li Z., Li J., Zou X., Yang X., Yang B., Zhang C., Wang H., Pui D. Y. H., Yang R. T. (2022). Separation of SO_2_ and NO_2_ with the Zeolite Membrane: Molecular Simulation Insights into the Advantageous NO_2_ Dimerization Effect. Langmuir.

[cit121] Von White G. , Tandon R., Serna L. M., Celina M. C. and Bernstein R., An Overview of Basic Radiation Effects on Polymers and Glasses, SAND2013-8003P, Sandia National Laboraties, Albuquerque, NM (United States), 2013.

[cit122] Georgii B. P., Gaponova I. S., Evgenii Ya D. (2000). Reactions of nitrogen oxides with polymers. Russ. Chem. Rev..

[cit123] Davydov E. Y., Gaponova I., Pokholok T., Vorotnikov A., Pariiskii G., Zaikov G. (2008). Features of mechanism of free radical initiation in polymers under exposure to nitrogen oxides. Polym. Adv. Technol..

[cit124] Baker R. W. , Membrane Technology and Applications, John Wiley & Sons, 2023.

[cit125] Sijbesma H., Nymeijer K., van Marwijk R., Heijboer R., Potreck J., Wessling M. (2008). Flue gas dehydration using polymer membranes. J. Membr. Sci..

[cit126] Merkel T., Baker R., Hao P., Kniep J., He Z., Huang Y., Salim W. (2026). Carbon capture with membranes: An opportunity revisited. J. Membr. Sci..

[cit127] Kim T.-J., Uddin M. W., Sandru M., Hägg M.-B. (2011). The effect of contaminants on the composite membranes for CO_2_ separation and challenges in up-scaling of the membranes. Energy Procedia.

[cit128] Sandru M., Kim T.-J., Capala W., Huijbers M., Hägg M.-B. (2013). Pilot Scale Testing of Polymeric Membranes for CO_2_ Capture from Coal Fired Power Plants. Energy Procedia.

[cit129] He X., Lindbråthen A., Kim T.-J., Hägg M.-B. (2017). Pilot testing on fixed-site-carrier membranes for CO_2_ capture from flue gas. Int. J. Greenhouse Gas Control.

[cit130] Deng L., Lindbråthen A., Janakiram S., Ansaloni L., Dai Z. (2024). Membranes and membrane processes for CO_2_ separation: MEMFO's long-term effort in reducing carbon emissions. Carbon Capture Sci. Technol..

[cit131] Scholes C. A., Chen G. Q., Stevens G. W., Kentish S. E. (2011). Nitric oxide and carbon monoxide permeation through glassy polymeric membranes for carbon dioxide separation. Chem. Eng. Res. Des..

[cit132] Haque M. F., Park S., Yang D. (2025). Gas sensors in harsh environments: Challenges and advances in high temperature, high humidity, radiative and corrosive conditions. J. Sci.:Adv. Mater. Devices.

[cit133] Croff A. , Wymer R., Tavlarides L., Flack J. and Larson H., status, and issues related to the regulation of advanced spent nuclear fuel recycle facilities NUREG-1909, US Nuclear Regulatory Commission, Washington, D. C, 2008.

[cit134] Arm S. T. , Comparison of Industrialized Late 20th Century Flowsheets for Reprocessing Used Nuclear Fuel, PNNL-38014, Pacific Northwest National Laboratory, Richland, WA, 2025.

[cit135] Hudson P. I. and Buckley C. P., Aerial and Liquid Effluent Treatment in BNFL's Thermal Oxide Reprocessing Plant (THORP) Japan, Japan Atomic Energy Research Institute, Tokyo (Japan), 1996.

[cit136] IAEA, Treatment of Radioactive Gaseous Waste, IAEA-TECDOC-1744, International Atomic Energy Agency, Vienna, Austria, 2014.

[cit137] Kawakatsu T. , Design and Construction of Rokkasho Reprocessing Plant, in Proceedings of the InternationalConference on Nuclear Fuel Reprocessing and Waste Management (RECOD), London, UK, 1994

[cit138] Dobson A. J. and Phillips C., High Level Waste Processing in the U.K. – Hard Won Experience that can Benefit U.S. Nuclear Cleanup Work, Proceedings of the Waste Management, Tucson, AZ, 2006.

[cit139] Liu Y., Zhou S., Wang Q., Luo Y., Wang F., Wang Z., Wang Y., Jin Y., Xia C. (2025). Xe/Kr adsorption and separation by homogeneous ultramicroporous polar activated carbon. Sep. Purif. Technol..

[cit140] Magomedbekov E. P., Merkushkin A. O., Obruchikov A. V., Pokalchuk V. S. (2022). Argon, krypton and xenon adsorption coefficients on various activated carbons under dynamic conditions. J. Radioanal. Nucl. Chem..

[cit141] Hong S.-M. (2025). Off-Gas Behavior and Capture Technologies in Pyroprocessing of Spent Nuclear Fuel. Korean J. Chem. Eng..

[cit142] Duong T. D., Li J., Li R., Lian X., Chen Y., Fan J., Hurd J., Guo L., Lee D., Warren M., Yang S. (2025). Efficient Capture and Separation of Xenon/Krypton from Off-Gas by a Radiation-Resistant Metal–Organic Framework. Small.

[cit143] Niu Z., Fan Z., Pham T., Verma G., Forrest K. A., Space B., Thallapally P. K., Al-Enizi A. M., Ma S. (2022). Self-Adjusting Metal–Organic Framework for Efficient Capture of Trace Xenon and Krypton. Angew. Chem., Int. Ed..

[cit144] Percival S. J., Small L. J., Bachman W. B., Schindelholz M. E., Nenoff T. M. (2023). Long-Term Durability and Cycling of Nanoporous Materials Based Impedance NO_2_ Sensors. Ind. Eng. Chem. Res..

[cit145] Sun Y., Yu Z., Zhang J., Xiao Y. (2025). Machine learning accelerated screening of zeolite structures for selective ammonia adsorption in mixed gas environments for automotive exhaust applications. Surf. Interfaces.

[cit146] Göltl F., Müller P., Uchupalanun P., Sautet P., Hermans I. (2017). Developing a Descriptor-Based Approach for CO and NO Adsorption Strength to Transition Metal Sites in Zeolites. Chem. Mater..

[cit147] Wang P., Xie K., Zhang C., Lin L. (2025). Enhanced Gas Sensing on Transition Metal-Doped PtSeTe: A Computational and Machine Learning Approach. Surf. Interfaces.

[cit148] Peng Z., Chen T., Dai L., Long M., Sun J. (2026). Machine Learning-Based Approach for Rapid Prediction and Analysis of Gas-Sensing Properties of Janus Materials. Langmuir.

