# Supplementary material for: Nitrogen oxides (NOx) in nuclear fuel reprocessing off-gas streams: chemistry, control and impacts on radionuclide capture
Source: RSC Adv. 2026 Nov 10. Online ahead of print. doi: 10.1039/d6ra08007c (PMC13750441; doi:10.1039/d6ra08007c)
Supplement: Supplementary file 1 [file RA-OLF-D6RA08007C-s001.pdf]

Supplementary Information (SI) for RSC Advances.
This journal is © The Royal Society of Chemistry

Supplemental Information

Nitrogen Oxides (NO_x) in Nuclear Fuel Reprocessing Off-Gas Streams: Chemistry, Control and Impacts on Radionuclide Capture

Jonathan Evarts[(a)*], Brian J. Riley[(a)], Stuart Arm[(a)], Katherine R. Johnson[(b)], Craig Milroy[(a)], Joanna McFarlane[(b)], Krista Carlson[(d)], Randy Ngelale[(b)], Richard T. Mayes[(b)], Laura Arias Chavez[(b)], Alexander Wiechert[(b)], Harmony S. Werth[(a)], Joshua Turner[(c)], Chase C. Cobble[(b)], and Jose Marcial[(a)]

[(a)] *Pacific Northwest National Laboratory, Richland, WA 99354, USA*
[(b)] *Oak Ridge National Laboratory, Oak Ridge, TN, 87830, USA*
[(c)] *National Nuclear Laboratory, Sellafield, UK*
[(d)] *Materials Science and Engineering, University of Nevada, Reno, Reno, NV, 89557, USA*

Table of Contents

S1 Additional References on Various Aspects Covered in the Paper *S3*
S2 NO_x Regulations and Exposure Limits *S5*
S3 NO_x Sources for Aqueous-Based UNF Reprocessing *S5*
S4 Radionuclide Inventory *S7*
S5 THORP *S9*
S6 NO_x Detection *S11*
S6.1 Chemiluminescence Methods S11
S6.2 Non-Dispersive Ultraviolet (NDIR) Absorption Methods S11
S6.3 Fourier Transform Infrared (FTIR) Spectroscopy Methods S11
S6.4 Tunable Diode Laser Absorption Spectroscopy (TDLAS) Methods S12
S6.5 Wet Chemical Methods S12
S6.6 Electrochemical Methods S13
S6.7 Ultraviolet Differential Optical Absorption Spectroscopy (UV-DOAS) Methods S13
S6.8 Mass Spectrometry Methods S14
S7 References *S14*

* Corresponding author: jonathan.evarts@pnnl.gov +1(509)372-7873

S1 Additional References on Various Aspects Covered in the Paper

Table S1. Additional References.

Topic	Section	Additional Refs.
Capture technologies for UNF off-gas streams	Section 1.0	[1]
Detrimental effects of using caustic scrubbers in off-gas capture streams	Section 1.0	[2,3]
Detrimental effects of NO_x streams on Ag-capture media on iodine capture	Section 1.0	[4]
NO_x abatement in industrial applications	Section 1.0	[5,6]
Industrial off-gas studies where NO_x concentrations are typically in the ppm range and contain fewer contaminates	Section 1.0	[7,8]
Decomposition of aqueous HNO_2	Section 3.1	[9]
Gas-phase oxidation of NO by HNO_3	Section 3.1	[10]
Presence of inorganic and organic iodine species have been documented in representative off-gas streams	Section 3.2	[11]
Pulverizing the fuel aids in subsequent dissolution	Section 4.0	[3,12-14]
Use of silver-based adsorbents for iodine capture	Section 5.1	[15,16]
Alternative metal (non-Ag) sorbents for iodine capture	Section 5.1	[17-19]
NO_x testing on sorbents	Section 5.1	[4,20-23]
MOFs for use as noble gas capture materials	Section 5.1	[24]
NO_x scrubbing with H_2O_2 with HNO_3	Section 6.1	[25]
NO_x scrubbing with $NaClO_2$ (sodium chlorite)	Section 6.1	[26-29]
NO_x scrubbing with porous solid sorbents	Section 6.2	[30,31]
NO_x scrubbing with catalytic transformation	Section 6.2	[32]
NO_x scrubbing with alkaline neutralization	Section 6.2	[33]
NO_x scrubbing by combining the sorbent with urea or carboxylic acid slurries	Section 6.2	[34]
Transition metal zeolites (Cu) for NO_x	Section 6.2	[35]
Metal oxides form nitrates species upon contact with NO_x	Section 6.2	[36]
TiO_2-V_2O_5 blends for NO_x capture	Section 6.2	[37]
NO_x removal from off gas of dissolvers or advanced head end processes	Section 6.3	[38,39]
Hanford LAW facility using SCR for NO_x	Section 6.3	[40]
Membrane separations with enhanced stability in organic solvents, acids, and higher temperatures	Section 6.4	[41-44]
Use of more radiolysis-resistant materials	Section 6.4	[45]
Recent advances for industrial separations	Section 6.4	[43,46]
Figure 5 data references.	Section 6.4	[47-51]

S2 NO_x Regulations and Exposure Limits

Table S2. Exposure limits and time for NO_x in an industrial environment derived from the NISOH pocket book[52] unless otherwise referenced.

Organization	Limit	Exposure Time	NO_2 Ceiling Limit (ppm)	NO Ceiling Limit [ppm]
OSHA	PEL	Apply Ceiling	5	25
ACIGH	TLV-TWA	8 hr average	0.2	25
ACGIH	STEL	15 min avg	1	n/a
NIOSH	REL	8 hr	1	25
NIOSH	IDLH	n/a	20	100
SCOEL[53]	OEL	8 hr average	0.5[53]	2[54]
SCOEL	STEL	15 min avg	1[53]	–

S3 NO_x Sources for Aqueous-Based UNF Reprocessing

Table S3. Assumptions for facility processing rate normalized to 1-MT batches for simplicity of comparison.

Facility Assumptions	
Batch Size	1 MT day^{-1}
Annual Uptime	200 days year^{-1}
Annual Rate	200 MT year^{-1}

Table S4. Species balance for an assumed 1 MT batch dissolution. NO_x generation rates derived from Marc and coworkers.[55]

Species	Compound	Moles/MTIHM
Reactants	UO_2	3,704
	HNO_3 initial	22,963
Products	$UO_2(NO_3)_2$	3,704
	NO	1,296
	NO_2	3,704
	H_2O (Generated)	6,111
H_2O	Initial	99,910
	Sum	106,021

S4 Radionuclide Inventory

Table S5. Selection of nuclides in a representative UNF inventory for an assumed 1 MT batch after 5-year cooling.

Radionuclide	Inventory (g/MTIHM)	Vapor Release (%)	Notes
Am-241	333.34	1E-03%	
Am-243	148.06	1E-03%	
Cm-244	54.18	1E-03%	
Cm-245	4.01	1E-03%	
Cs-134	34.45	1E-03%	Aerosol[56]
Cs-135	480.62	1E-03%	
Cs-137	1,475.60	1E-03%	
Eu-154	22.73	1E-03%	
Eu-155	5.51	1E-03%	
I-129	200.56	99%	Volatile[57]
Kr-85	24.12	100%	Volatile[58]
Nd-144	1,889.00	1E-03%	
Np-237	572.26	1E-03%	
Pd-107	286.79	1E-03%	
Pm-147	63.48	1E-03%	
Pu-238	258.79	1E-03%	Aerosol[56]
Pu-239	5,388.10	1E-03%	
Pu-240	2,721.70	1E-03%	
Pu-241	1,087.90	1E-03%	
Pu-242	690.70	1E-03%	
Ru-106	6.96	<0.10% + 1E-03%	Semi-volatile[59] Aerosol[56]
Sb-125	3.31	1E-03%	
Se-79	6.14	1E-03%	
Sm-147	268.48	1E-03%	
Sm-148	192.33	1E-03%	
Sm-151	12.74	1E-03%	
Sn-126	26.95	1E-03%	
Sr-90	654.04	1E-03%	Aerosol[56]
Tc-99	1,067.20	1E-03%	
U-234	207.60	1E-03%	
U-235	8,604.30	1E-03%	
U-236	5,765.10	1E-03%	
U-238	927,410.00	1E-03%	
Zr-93	970.45	1E-03%	
SUM	9.61×10^{05}		

S5 THORP

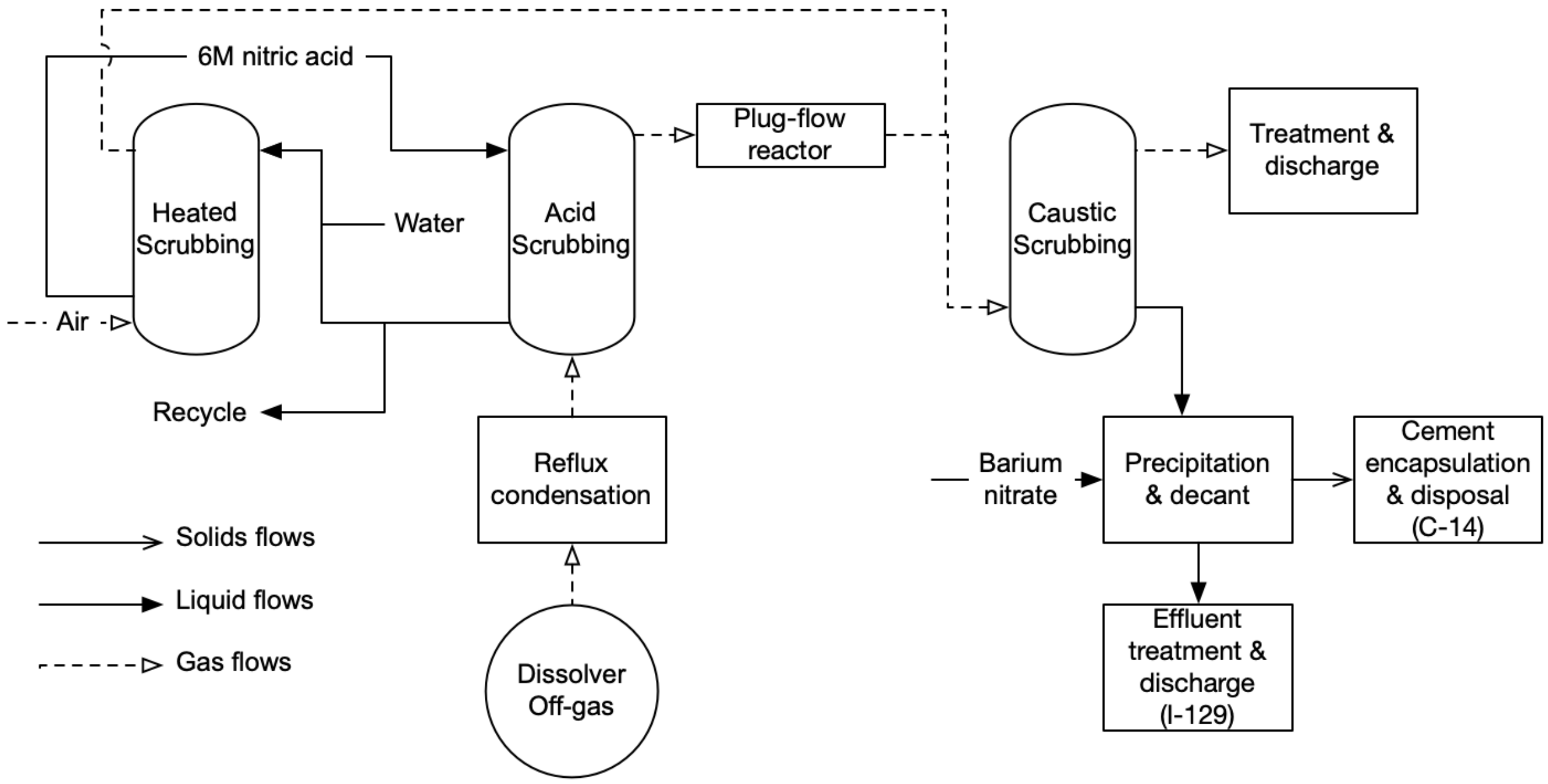

Figure S1. THORP reprocessing flowchart for dissolver off-gas treatment.

Substantial experimental work at a pilot scale of the DOG system was carried out during the development of Thorp and this data was used to create a process model for the NO_x behavior in the off-gas system.[60] This model was then used to inform the design of the central off-gas (COG) system. The COG system managed most of the other process vessel off gas downstream of the dissolver. It worked on the principle of combining different off-gas streams based on their off-gas challenge, so that targeted abatement could take place. Whilst the DOG flow rates were 400 m^3/hr, the COG could be an order of magnitude higher during operational periods (up to 8000 m^3/hr), which had a consequence on the DFs that could be achieved in these process vessels. The main NO_x sources in the COG were from salt free evaporation, nitric acid recovery, salt azide conditioning and plutonium purification processes. Of these, the SFE, NAR and SAC also discharged iodine although relatively at a lower quantity relative to the DOG.

The thermal denitrator in the uranium finishing part of Thorp converted a concentrated solution of uranyl nitrate into uranyl oxide which had the side effect of releasing NO_x [eq. (S1)]. This was then abated with condenser and a caustic scrubber as shown in Equation (S1). The gloveboxes involved with the THORP product

finishing line, plutonium purification, decontamination and HA/UP cycle areas had the potential to release NO_x which was unabated, however levels were low compared to other sources of discharge.[61]

$$UO_2(NO_3)_2 + nH_2O \rightarrow UO_3 + 2NO_x + nH_2O + O_2 \tag{S1}$$

In addition to Thorp, significant NO_x arisings from downstream plants such as the waste vitrification plant.[62,63] Here a nitric acid liquor containing the highly active fission products is fed through a calciner before being vitrified. The evaporation of the nitric acid as well as the denitration of the fission products leads to the production of NO_x. This is abated in a series of acidic scrubbers, the dust scrubber, condenser and NO_x absorber al. designed to achieve different functions, but each contribute to minimizing NO_x discharges.[63] Other facilities that handled nitric acid liquors also were expected to discharge NO_x, but in much smaller quantities such as in the HALES, STP and LAEMG facilities. Table S6 (SI) provides a summary of the processes and facilities in THORP that discharge NO_x and the corresponding off-gas abatement units that prevent NO_x release.

Table S6. Summary of Off-gas systems relevant to NO_x abatement

**Off-gas System**	**NO_x Release**	**Abatement Method**
DOG	• Dissolution of nuclear fuel<br>• NO_x sparge	Condenser, Acid Recombination Column, Plug Flow reactor, Caustic Scrubber
COG	• Pu purification<br>• Salt azide conditioning<br>• Salt free evaporation<br>• Nitric acid recovery	Caustic Scrubber
Thermal denitration	• Conversion of uranyl nitrate to uranium oxide	Acid condenser, Caustic Scrubber
Finishing line gloveboxes	N/A	N/A
Waste Vitrification Plant	• Evaporation, calcination and vitrification of highly active liquor	Acidic Scrubbing
HALES	• Evaporation of HNO_3 liquors<br>• Radiolysis during storage of highly active liquors	Acidic Scrubber, Caustic Scrubber

S6 NO_x Detection

S6.1 Chemiluminescence Methods

Chemiluminescence is the most common method for NO_x detection. Decades of work on detectors of this type have developed this technology and it is widely available.[64,65] This method distinguishes between NO and NO_2 a two-step process. First, the NO is reacted with ozone and the light produced by the reaction is measured. Second, NO_2 is converted to NO, typically with a heated molybdenum catalytic converter, which is then also reacted with ozone to measure the light produced. The difference in measurement allows for determination of NO and NO_2 concentrations. Advantages of this method are high sensitivity and fast, linear responses. However, catalytic conversion of NO_2 may suffer from conversion inefficiencies and interferences from other nitrogen compounds during conversion, leading to a lower or higher measurement than the true concentration, respectively.[66]

S6.2 Non-Dispersive Ultraviolet (NDIR) Absorption Methods

Non-dispersive sensing techniques are typically rely low cost and compact instruments.[67] NDIR gas detection is achieved by shining infrared light through the gas and measuring the amount of light absorbed at specific wavelengths compared to the target gas. The intensity drop is used to determine gas concentration according to the Beer-Lamber law. Instrument design (spectral filters, path length, light source, detector type, etc.) has a major impact on detection capabilities, so systems are typically designed for the detection of a single target species.[68] Xu et al. constructed a NO_x NDIR detection system and achieved detection in the ranges of 0 – 5000 ppm for NO and 0 – 1000 ppm for NO_2.[69] Limitations of NDIR systems include are sensitivity to temperature and pressure that can cause calibration errors. Additionally, water vapor as well as other gas components may interfere with measurement of the target gas.

S6.3 Fourier Transform Infrared (FTIR) Spectroscopy Methods

Fourier transform infrared (FTIR) spectroscopy is a full-spectrum, high resolution technique capable of analyzing and distinguishing between several target gases simultaneously, including NO and NO_2. For FTIR, IR light enters a Michelson interferometer, which contains a beamsplitter and a configuration of mirrors. One of the

mirrors moves, causing wave interference to generate a different spectrum exiting the interferometer. A Fourier transform converts the data collected for light absorption for each mirror position into an absorption spectrum, which can then be interpreted to determine gas species and concentration. Depending on the application, a low-resolution FTIR with a simplified design and lower cost may be used for NO_x real-time monitoring.[70] FTIR has also been investigated for online monitoring of NO evolution with good agreement with other monitoring methods.[71] As with other spectroscopic gas analysis techniques, a potential weakness of FTIR is interference in NO_x measurement by other gases with overlapping IR bands.

S6.4 Tunable Diode Laser Absorption Spectroscopy (TDLAS) Methods

Two types of tunable diode laser absorption spectroscopy (TDLAS), direct spectroscopy (DS) and wavelength modulation spectroscopy (WMS) are widely available and have well-established techniques for trace gas measurement.[72,73] For this technique, a tunable diode laser is swept across an absorption line of the target species and the light travels through the sample gas. The intensity reduction due to absorption is measured with a photodetector and used to determine the target gas concentration. The equipment for TDLAS is relatively expensive and can only detect one kind of gas at a time. However, since the laser linewidth is extremely narrow ($\sim 10^{-3}$ cm^{-1}), this method can distinguish between NO and NO_2 and can achieve extremely low detection limits. Improvements in laser diode technology and the selection of absorption lines has continued to improve TDLAS sensitivity.[74] Rao et al.[75] have achieved a NO_x detection limit as low as 28 ppt by utilizing a quantum cascade laser.

S6.5 Wet Chemical Methods

Various wet chemical methods typically rely on gas absorption into a liquid, conversion of NO_x gas to nitrite/nitrate, then quantification by methods such as titration, colorimetry, or other analytical methods. Common methods include the Griess-Saltzman method and the Jacobs-Hochheiser method.[76-78] Wet chemical methods often require NO to oxidize to NO_2, resulting in total NO_x concentration measurement. In the Griess-Saltzman method, the gas of interest is bubbled through a reagent which consists of sulfanilic acid, glacial acetic acid, and *N*-(1-naphthyl)-ethylenediamine dihydrochloride. The result is a color change that is highly specific for NO_2.[78] Typically, wet chemical methods use techniques such as colorimetry or ion chromatography to quantify

NO_x concentration. Several variations have advanced upon the technique for different applications, such as combining it with RGB sensors for rapid analysis of 50 ppb to 300 ppm NO_2.[79]

S6.6 Electrochemical Methods

Electrochemical sensors for NO_x detection have been well studied, with several options for detection mechanisms and materials.[80-82] Electrochemical sensors are well-suited for high temperature environments and typically provide rapid sensing signals and good selectivity for the target gas, although they have a limited lifetime and require regular calibration for accurate measurement. Electrochemical gas sensors consist of a gas permeable membrane as well as a sensing electrode, working electrode, counter electrode, and reference electrode. The membrane is in contact with the surrounding atmosphere, allowing NO_x to diffuse through the membrane. The gas reduces or oxidizes at the surface of the sensing electrode, causing a current flow between the sensing and working electrode and resulting in an electrical signal that can be interpreted to determine gas concentration.[80] Electrochemical sensors can be designed to generate responses to the target species related to voltage (potentiometric), current (amperometric), or resistance (impedancemetric).[81] Of these types, amperometric sensors are the dominant type for NO_x measurement due to the linear current response for detection, leading to accurate real time measurements for ppm levels.[81,83] A challenge for amperometric sensors is selectivity since any species containing the mobile ion can contribute to the current. Yttria-stabilized zirconia (YSZ) is a common choice for a solid electrolyte due to its thermal and chemical stability as well as high oxygen ion conductivity. Electrode materials may be pure gold or platinum, or materials specialized for improved sensitivity such as $La_{0.8}Sr_{0.2}MnO_3$ (LSM).

S6.7 Ultraviolet Differential Optical Absorption Spectroscopy (UV-DOAS) Methods

Differential optical absorption spectroscopy has commonly been used for monitoring of pollutants, including NO_x, in the atmosphere.[84] For DOAS measurement, a light passes through the gas in an adsorption cell and measurement of the species of interest is based on the difference between local maximum and minima in the absorption spectrum of the gas.[85] UV light is used for NO_x detection as NO and NO_2 have absorption bands in the UV range.[67] Some of the advantages of the techniques is that it is non-destructive, selective, and capable of detecting multiple components simultaneously. However, another gas species may overlap with NO_x absorption

bands, which complicates NO_x measurement, especially at low concentrations. SO_2, for instance, has an absorption spectrum that completely overlaps with that of NO.[86] Spectrum analysis techniques and experimental setup may be devised to achieve detection even in mixed gas environments, such as in work by Xie et al. that achieved a detection limit for NO of 2.53 ppb.[67]

S6.8 Mass Spectrometry Methods

Gas analysis by mass spectrometry requires the creation of a charged ion from the sample gas and measures the mass-to-charge ratio to determine the species. A variety of analyzer types are available based on the intended application: quadrupole-based systems (small, fast, and inexpensive); sector-based systems (stable but slow); time-of-flight systems (TOF) that are more sensitive, but larger in size; and dual-focusing sector systems, which are best when high precision with a small gas volume are required; as well as other types.[87] Systems may also be described by whether they are used for static or continuous gas monitoring. Although NO_x detection mass spectrometry is not affected by overlapping absorption bands as with spectroscopic techniques, gas molecules of a similar m/z will interfere with accurate measurement. For NO_x measurement, interferences from numerous parent and fragment ion peaks make reliable NO and NO_2 detection impossible when using electron ionization.[88] As a result, specialized analysis or specific instrument selection are necessary for simultaneous NO and NO_2 analysis. Bornschelgl et al.[88] has achieved NO and NO_2 simultaneous monitoring with a combination of laser spectroscopy and a TOF system. A broad bandwidth resonance enhanced multiphoton ionization (REMPI) mass spectroscopy has been used by McKeashie et al.[89] for NO and NO_2 detection.

S7 References

[1] M. Greenhalgh, A. Welty, M.S. Fujimoto, K. Johnson, L. Martin, R. Mayes, P.K. Thallapally, B.J. Riley. 2024. PNNL-36165; INL/RPT-24-79059. Pacific Northwest National Laboratory, Richland, WA.

[2] G. Chen, L. Wu, Z. Wang, R. Liu, C. Tan, Y. Tan, Y. Liu, M. He, H. Zhang, Y. Huang, J. Zhu, T. Duan, L. Zhu, Aluminum-based MOF for efficient iodine and methyl iodide capture from NO_2-containing gas streams, *Chem. Eng. J.*, 2025, **507**, 160829.

[3] G.D. Del Cul, J.A. Johnson, B.B. Spencer, E.D. Collins. 2013. "Roadmap for development of an advanced head-end reactor" *In Proc. of the GLOBAL 2013*. pp. 879–881. American Nuclear Society.

[4] K.K. Patton, S.H. Bruffey, J.T. Jubin, J. Walker, J. F. 2014. ORNL/LTR-2014/42. Oak Ridge National Laboratory, Oak Ridge, TN.

[5] X. Guimerà, M. Mora, A.D. Dorado, A. Bonsfills, D. Gabriel, X. Gamisans, Optimization of SO_2 and NO_x sequential wet absorption in a two-stage bioscrubber for elemental sulphur valorisation, *Environ. Sci. Pollut. Res.*, 2021, **28**, 24605–24617.

[6] Y.S. Mok, H.-J. Lee, Removal of sulfur dioxide and nitrogen oxides by using ozone injection and absorption–reduction technique, *Fuel Proc. Technol.*, 2006, **87**, 591–597.

[7] N. Bion, F. Can, X. Courtois, D. Duprez, "Transition metal oxides for combustion and depollution processes," pp. 287–353. In *Metal Oxides in Heterogeneous Catalysis.* Edited by J. C. Védrine. Elsevier, 2018.

[8] Z. Chen, Q. Liu, H. Liu, T. Wang, Recent Advances in SCR Systems of Heavy-Duty Diesel Vehicles—Low-Temperature NO_x Reduction Technology and Combination of SCR with Remote OBD, *Atmosphere*, 2024, **15**.

[9] E. Kaiser, C. Wu, A kinetic study of the gas phase formation and decomposition reactions of nitrous acid, *J. Phys. Chem.*, 1977, **81**, 1701–1706.

[10] C.E. Corriveau, "The Absorption of N_2O_3 Into Water," Master's Thesis, University of California, Berkeley, 1971.

[11] S.H. Bruffey, B.B. Spencer, D.M. Strachan, R.T. Jubin, N.R. Soelberg, B.J. Riley. 2015. INL/EXT-15-35609. Idaho National Laboratory, Idaho Falls, ID.

[12] J.-J. Park, C.-J. Park, J. Chun, J. Lee, J. Shin, G.-I. Park, K.-C. Song, "Evaluation of the effects of the advanced voloxidation process on pyro-processing." In *Radiation and decay heat analysis of the advanced voloxidation process*. Daejeon: KAERI, 2008.

[13] R. Jubin. 2008. "Spent Fuel Reprocessing" *In Proc. of the Introduction to Nuclear Chemistry and Fuel Cycle Separations Course*. Consortium for Risk Evaluation with Stakeholder Participation (CRESP).

[14] A.T. Greaney, R.O. Ngelale, S.H. Bruffey, L.R. Martin, Abatement of radioiodine in aqueous reprocessing off-gas, *Front. Chem.*, 2023, **10**, 1078668.

[15] S. Chong, B.J. Riley, J.A. Peterson, M.J. Olszta, Z.J. Nelson, Gaseous Iodine Sorbents: A Comparison between Ag-Loaded Aerogel and Xerogel Scaffolds, *ACS Appl. Mater. Interfaces*, 2020, **12**, 26127–26136.

[16] L. Liao, S. Song, G. Liang, X. Wang, N. Pan, H. Lei, Y. Zhang, H. Zou, J. Cheng, J. Wen, W. Zhang, T. Deng, Resource recovery from iodine-containing silver-loaded silica gel: Recycling of silver and immobilization of iodine, *J. Haz. Mater.*, 2025, **493**, 138306.

[17] Y.E. Jung, J.H. Yang, M.-S. Yim, Investigation of bismuth-based metal-organic frameworks for effective capture and immobilization of radioiodine gas, *J. Haz. Mater.*, 2024, **467**, 133777.

[18] J. Zhou, T. Lan, T. Li, Q. Chen, P. Bai, F. Liu, Z. Yuan, W. Zheng, X. Luo, W. Yan, T. Yan, Highly efficient capture of iodine in spent fuel reprocessing off-gas by novelly porous copper-doped silica zeolites, *Separ. Purif. Technol.*, 2022, **290**, 120895.

[19] X. Liang, G. Chen, H. Zhang, L. Zhang, T. Duan, L. Zhu, Co-adsorption performance of iodine and NO_x in iodine exhaust gas by NH_2-MIL-125, *J. Haz. Mater.*, 2024, **470**, 134237.

[20] S. Choi, Y. Nan, L.L. Tavlarides, Kinetics of aging process on reduced Ag exchanged mordenite in dry air and humid air, *AIChE J.*, 2021, **67**, e17182.

[21] K.K. Patton, S.H. Bruffey, J.F. Walker, R.T. Jubin. 2014. ORNL/LTR-2014/271. Oak Ridge National Laboratory, Oak Ridge, TN.

[22] S.H. Bruffey, K.K. Anderson, J.F. Walker Jr., R.T. Jubin. 2013. FCRD-SWF-2013-000258. Oak Ridge National Laboratory, Oak Ridge, TN.

[23] J. Matyáš, E.S. Ilton, L. Kovařík, Silver-functionalized silica aerogel: towards an understanding of aging on iodine sorption performance, *RSC Adv.*, 2018, **8**, 31843–31852.

[24] J. Pei, X.-W. Gu, C.-C. Liang, B. Chen, B. Li, G. Qian, Robust and Radiation-Resistant Hofmann-Type Metal–Organic Frameworks for Record Xenon/Krypton Separation, *J. Am. Chem. Soc.*, 2022, **144**, 3200–3209.

[25] S. Kartohardjono, C. Merry, M.S. Rizky, C.C. Pratita, Nitrogen oxide reduction through absorbent solutions containing nitric acid and hydrogen peroxide in hollow fiber membrane modules, *Heliyon*, 2019, **5**, e02987.

[26] E. Sada, H. Kumazawa, I. Kudo, T. Kondo, Absorption of NO in aqueous mixed solutions of $NaClO_2$ and NaOH, *Chem. Eng. Sci.*, 1978, **33**, 315–318.

[27] E. Sada, H. Kumazawa, Y. Yamanaka, I. Kudo, T. Kondo, Kinetics of absorption of sulfur dioxide and nitric oxide in aqueous mixed solutions of sodium chlorite and sodium hydroxide, *J. Chem. Eng. Jpn*, 1978, **11**, 276–282.

[28] C. Brogren, H.T. Karlsson, I. Bjerle, Absorption of NO in an Aqueous Solution of $NaClO_2$, *Chem. Eng. Technol.*, 1998, **21**, 61–70.

[29] H.W. Hsu, C.J. Lee, K.S. Chou, Absorption of NO by $NaClO_2$ solution: Performance characteristics, *Chem. Eng. Comm.*, 1998, **170**, 67–81.

[30] Y. Liu, X. Wu, X. Yang, H. Tao, J. Li, C. Zhang, R.T. Yang, Z. Li, Enhancement of NO_x adsorption performance on zeolite via a facile modification strategy, *J. Haz. Mater.*, 2023, **443**, 130225.

[31] K. Skalska, J.S. Miller, S. Ledakowicz, Trends in NO_x abatement: A review, *Sci. Total Environ.*, 2010, **408**, 3976–3989.

[32] X. Cheng, X.T. Bi, A review of recent advances in selective catalytic NO_x reduction reactor technologies, *Particuology*, 2014, **16**, 1–18.

[33] X. Zhang, H. Tong, H. Zhang, C. Chen, Nitrogen Oxides Absorption on Calcium Hydroxide at Low Temperature, *Ind. Eng. Chem. Res.*, 2008, **47**, 3827–3833.

[34] B.K. Gullett, K.R. Bruce, W.F. Hansen, J.E. Hofmann, Sorbent/Urea slurry injection for simultaneous SO_2/NO_x removal, *Environ. Prog.*, 1992, **11**, 155–162.

[35] X. Ye, R. Oord, M. Monai, J.E. Schmidt, T. Chen, F. Meirer, B.M. Weckhuysen, New insights into the NH_3-selective catalytic reduction of NO over Cu-ZSM-5 as revealed by operando spectroscopy, *Catal. Sci. Technol.*, 2022, **12**, 2589–2603.

[36] J. Yu, F. Guo, J. Yang, Y. Wang, L. Dong, S. Gao, G. Xu, "Surface deposition-type honeycomb catalyst for flue gas denitrification and preparation method thereof," patent application number US Patent No. 9,446,385, US Patent No. 9,446,385, 2016.

[37] X. Zhao, L. Huang, H. Li, H. Hu, J. Han, L. Shi, D. Zhang, Highly dispersed V_2O_5/TiO_2 modified with transition metals (Cu, Fe, Mn, Co) as efficient catalysts for the selective reduction of NO with NH_3, *Chinese J. Catal.*, 2015, **36**, 1886–1899.

[38] K.S. Matlack, W. Gong, T. Bardakci, N. D'Angelo, M. Brandys, W.K. Kot, I.L. Pegg, A. Kruger. 2011. ORP-51444-Rev-0; VSL-05R5830-1. USDOE Office of River Protection, Richland, WA.

[39] L. Lewis, A. Kruger. 2015. 24590-CD-POC-MBT0-00007-02-00001_00E. Hanford Waste Treatment and Immobilization Plant, Richland, WA.

[40] A. Goel, J.S. McCloy, R. Pokorny, A.A. Kruger, Challenges with vitrification of Hanford High-Level Waste (HLW) to borosilicate glass – An overview, *J. Non-Cryst. Solids*, 2019, **4**, 100033.

[41] R. Thiruvenkatachari, M. Manickam, T. Ouk Kwon, I. Shik Moon, J. Woo Kim, Separation of Water and Nitric Acid with Porous Hydrophobic Membrane by Air Gap Membrane Distillation (AGMD), *Separ. Sci. Technol.*, 2006, **41**, 3187–3199.

[42] C. Ponce-de-León, R.W. Field, Comparison of anionic membranes used to concentrate nitric acid to beyond the azeotropic mixture, *J. Membr. Sci.*, 2000, **171**, 67–77.

[43] S.P. Nunes, P.Z. Culfaz-Emecen, G.Z. Ramon, T. Visser, G.H. Koops, W. Jin, M. Ulbricht, Thinking the future of membranes: Perspectives for advanced and new membrane materials and manufacturing processes, *J. Membr. Sci.*, 2020, **598**, 117761.

[44] A. Oxley, A.G. Livingston, Effect of polymer molecular weight on the long-term process stability of crosslinked polybenzimidazole organic solvent nanofiltration (OSN) membranes, *J. Membr. Sci.*, 2024, **689**, 122149.

[45] A. Bedar, R.K. Lenka, N.K. Goel, S. Kumar, R.D. Jain, B.G. Singh, P.K. Tewari, R.C. Bindal, S. Kar, Enhancement of γ-radiation stability of polysulfone membrane matrix by reinforcement of hybrid nanomaterials of nanodiamond and ceria, *Mater. Adv.*, 2020, **1**, 1220–1231.

[46] H.B. Park, J. Kamcev, L.M. Robeson, M. Elimelech, B.D. Freeman, Maximizing the right stuff: The trade-off between membrane permeability and selectivity, *Science*, 2017, **356**, eaab0530.

[47] N.H. Heo, K.H. Cho, J.T. Kim, K. Seff, Crystal Structures of Encapsulates within Zeolites. 1. Krypton in Zeolite A, *J. Phys. Chem.*, 1994, **98**, 13328–13333.

[48] G.-J. Hwang, K. Onuki, Simulation study on the catalytic decomposition of hydrogen iodide in a membrane reactor with a silica membrane for the thermochemical water-splitting IS process, *J. Membr. Sci.*, 2001, **194**, 207–215.

[49] H. Kalipcilar, T.C. Bowen, R.D. Noble, J.L. Falconer, Synthesis and Separation Performance of SSZ-13 Zeolite Membranes on Tubular Supports, *Chem. Mater.*, 2002, **14**, 3458–3464.

[50] Y. Liu, J. Zuo, Z. Li, J. Li, X. Zou, X. Yang, B. Yang, C. Zhang, H. Wang, D.Y.H. Pui, R.T. Yang, Separation of SO2 and NO2 with the Zeolite Membrane: Molecular Simulation Insights into the Advantageous NO2 Dimerization Effect, 2022, **38**, 2751–2762.

[51] R.D. Scheele, L.L. LBurger, C.L. Matsuzaki. 1983. PNL-4489. Pacific Northwest National Laboratory, Richland, WA.

[52] N.A. Cdc, "NIOSH pocket guide to chemical hazards," 2018.

[53] SCOEL. 2014. SCOEL/SUM/53. European Commission.

[54] SCOEL. 2014. SCOEL/SUM/89. European Commission.

[55] P. Marc, A. Magnaldo, A. Vaudano, T. Delahaye, É. Schaer, Dissolution of uranium dioxide in nitric acid media: what do we know?, *EPJ Nuclear Sci. Technol.*, 2017, **3**, 13.

[56] F.J. Herrmann, V. Motoi, B. Herrmann, A. Van Schoor, D. Fang, H. Fies, Retention and Measurement of Iodine-129 and of Organoiodine in the Offgas Streams of the Karlsruhe Reprocessing Plant WAK, *Proceedings of the 21st DOE/NRC Nuclear Air Cleaning Conference*, 1990, **1**, 222–233.

[57] R.T. Jubin, D.M. Strachan, N.R. Soelberg. 2013. INL/EXT-13-30119. Idaho National Laboratory, Idaho Falls, ID.

[58] J.D. Vienna, E.D. Collins, J.V. Crum, W.L. Ebert, S.M. Frank, T.G. Garn, D. Gombert, R. Jones, R.T. Jubin, V.C. Maio, J.C. Marra, J. Matyas, T.M. Nenoff, G.J. Sevigny, N.R. Soelberg, D.M. Strachan, P.K. Thallapally, J.H. Westsik. 2015. PNNL-24114. Pacific Northwest National Laboratory, Richland, WA.

[59] B.B. Spencer, R.T. Jubin, S.H. Bruffey, D.M. Strachan. 2017. NTRD-MRWFD-2017-000312; ORNL/TM-2017/330. Oak Ridge National Laboratory, Oak Ridge, TN.

[60] P.I. Hudson, C.P. Buckley, *Aerial and liquid effluent treatment in BNFL's Thermal Oxide Reprocessing Plant (THORP).* Japan: ; Japan Atomic Energy Research Inst., Tokyo (Japan) 1996.

[61] P.I. Hudson, C.P. Buckley, W.W. Miller. 1995. "The development and design of the off-gas treatment system for the thermal oxide reprocessing plant (THORP) at Sellafield" *In Proc. of the 23rd DOE/NRC*

Nuclear Air Cleaning and Treatment Conference. pp. CONF–940738. Harvard Air Cleaning Laboratory.

[62] M.T. Harrison, Vitrification of High Level Waste in the UK, *Proced. Mater. Sci.*, 2014, **7**, 10–15.

[63] A. Riley, S. Walker, N.R. Gribble, Composition Changes and Future Challenges for the Sellafield Waste Vitrification Plant, *MRS Proc.*, 2009, **1193**, 267.

[64] J.W. Drummond, A. Volz, D.H. Ehhalt, An optimized chemiluminescence detector for tropospheric NO measurements, *J. Atmos. Chem.*, 1985, **2**, 287–306.

[65] K.H. Becker, W. Groth, D. Thran, The mechanism of the air-afterglow NO + O → NO_2 + hν, *Chem. Phys. Lett.*, 1972, **15**, 215–220.

[66] D.R. Worton, Future Adoption of Direct Measurement Techniques for Regulatory Measurements of Nitrogen Dioxide: Drivers and Challenges, *Environ. Sci. Technol.*, 2020, **54**, 14785–14786.

[67] F. Xie, Y. Wu, J. Gao, M. Li, R. Zhu, X. Wu, Y. Zhang, Detection of ultra-low concentration NH_3, SO_2, and NO using UV-DOAS combined with multidimensional spectral fusion, *Fuels*, 2024, **378**, 132965.

[68] J. Hodgkinson, R.P. Tatam, Optical gas sensing: a review, *Meas. Sci. Technol.*, 2013, **24**, 012004.

[69] M. Xu, C. Gao, Y. Guo, Design of nitrogen oxide detection system based on non-dispersive infrared technology, *Optik*, 2022, **262**, 169351.

[70] K.T. Larjava, K.E. Tormonen, P.T. Jaakkola, A.A. Roos, Field Measurements of Flue Gases from Combustion of Miscellaneous Fuels Using a Low-Resolution FTIR Gas Analyzer, *J. Air Waste Manag. Assoc.*, 1997, **47**, 1284–1290.

[71] A. Sánchez, E. Eddings, F. Mondragón, Fourier Transform Infrared (FTIR) Online Monitoring of NO, N_2O, and CO_2 during Oxygen-Enriched Combustion of Carbonaceous Materials, *Energy Fuels*, 2010, **24**, 4849–4853.

[72] M.W. Sigrist, R. Bartlome, D. Marinov, J.M. Rey, D.E. Vogler, H. Wächter, Trace gas monitoring with infrared laser-based detection schemes, *Appl. Phys. B*, 2008, **90**, 289–300.

[73] P. Werle, F. Slemr, K. Maurer, R. Kormann, R. Mücke, B. Jänker, Near- and mid-infrared laser-optical sensors for gas analysis, *Opt. Lasers Eng.*, 2002, **37**, 101–114.

[74] S. Lin, J. Chang, J. Sun, P. Xu, Improvement of the Detection Sensitivity for Tunable Diode Laser Absorption Spectroscopy: A Review, *Front. Phys.*, 2022, **Volume 10**, 853966.

[75] G.N. Rao, A. Karpf, Extremely sensitive detection of NO2 employing off-axis integrated cavity output spectroscopy coupled with multiple-line integrated absorption spectroscopy, *Appl. Opt.*, 2011, **50**, 1915–1924.

[76] M.B. Jacobs, S. Hochheiser, Continuous sampling and ultramicrodetermination of nitrogen dioxide in air, *Anal. Chem.*, 1958, **30**, 426–428.

[77] T.A.-O. Chin, I.C. Tam, C.Y. Yin, Comparison of various chemical compounds for the removal of SO_2 and NO_x with wet scrubbing for marine diesel engines, *Env. Sci. Pollut. Res.*, 2022, **29**, 8873–8891.

[78] B.E. Saltzman, Colorimetric Microdetermination of Nitrogen Dioxide in Atmosphere, *Anal. Chem.*, 1954, **26**, 1949–1955.

[79] C. Fàbrega, L. Fernández, O. Monereo, A. Pons-Balagué, E. Xuriguera, O. Casals, A. Waag, J.D. Prades, Highly Specific and Wide Range NO_2 Sensor with Color Readout, *ACS Sensors*, 2017, **2**, 1612–1618.

[80] M.A. Khan, F. Qazi, Z. Hussain, M.U. Idrees, S. Soomro, S. Soomro, Recent Trends in Electrochemical Detection of NH_3, H_2S and NO_x Gases, *Int. J. Electrochem. Sci.*, 2017, **12**, 1711–1733.

[81] J.W. Fergus, Materials for high temperature electrochemical NO_x gas sensors, *Sens. and Actuators B: Chem.*, 2007, **121**, 652–663.

[82] F. Menil, V. Coillard, C. Lucat, Critical review of nitrogen monoxide sensors for exhaust gases of lean burn engines, *Sens. Actuators B: Chem.*, 2000, **67**, 1–23.

[83] M. Aliramezani, C.R. Koch, M. Secanell, R.E. Hayes, R. Patrick, An electrochemical model of an amperometric NO- sensor, *Sens. Actuators B Chem.*, 2019, **290**, 302–311.

[84] J. Mellqvist, A. Rosén, DOAS for flue gas monitoring—I. Temperature effects in the UV/visible absorption spectra of NO, NO_2, SO_2 and NH_3, *J. Quant. Spectrosc. Radiat. Transf.*, 1996, **56**, 187–208.

[85] U. Platt, J. Stutz, "Differential absorption spectroscopy," pp. 135–174. In *Differential Optical Absorption Spectroscopy: Principles and Applications*. Edited by U. Platt and J. Stutz. Springer, Heidelberg, Germany, 2008.

[86] B. Peng, Y. Zhou, G. Liu, Y. He, C. Gao, Y. Guo, An ultra-sensitive detection system for sulfur dioxide and nitric oxide based on improved differential optical absorption spectroscopy method, *Spectrochim. Acta A Mol. Biomol. Spectrosc.*, 2020, **233**, 118169.

[87] C.D. Mowry, R.L. Jarek, J. Kustas, A.C. Telles, A.S. Pimentel, "Gas Analysis by Mass Spectrometry," pp. 143–152. In *ASM Handbook, Materials Characterization*, **Vol. 10**. Sandia National Laboratories, Albuquerque, NM 2020.

[88] A. Bornschlegl, R. Weishaeupl, U. Boesl, A new approach for fast, simultaneous NO/NO_2 analysis: application of basic features of multiphoton-induced ionization and dissociation of NO_x, *Anal. Bioanal. Chem.*, 2006, **384**, 161–168.

[89] J.R. McKeachie, W.E. van der Veer, L.C. Short, R.M. Garnica, M.F. Appel, T. Benter, Selective ultra-trace detection of NO and NO_2 in complex gas mixtures using broad-bandwidth REMPI mass spectrometry, *Analyst*, 2001, **126**, 1221–1228.
